# Anticancer Properties of Platinum Nanoparticles and Retinoic Acid: Combination Therapy for the Treatment of Human Neuroblastoma Cancer

**DOI:** 10.3390/ijms21186792

**Published:** 2020-09-16

**Authors:** Sangiliyandi Gurunathan, Muniyandi Jeyaraj, Min-Hee Kang, Jin-Hoi Kim

**Affiliations:** Department of Stem Cell and Regenerative Biotechnology, Konkuk University, Seoul 05029, Korea; muniyandij@yahoo.com (M.J.); pocachippo@konkuk.ac.kr (M.-H.K.)

**Keywords:** anticancer activity, platinum nanoparticle, retinoic acid, cytotoxicity, oxidative stress, endoplasmic reticulum stress, mitochondrial dysfunctions, apoptosis

## Abstract

Neuroblastoma is the most common extracranial solid tumor in childhood. The different treatments available for neuroblastoma are challenged by high rates of resistance, recurrence, and progression, most notably in advanced cases and highly malignant tumors. Therefore, the development of more targeted therapies, which are biocompatible and without undesired side effects, is highly desirable. The mechanisms of actions of platinum nanoparticles (PtNPs) and retinoic acid (RA) in neuroblastoma have remained unclear. In this study, the anticancer effects of PtNPs and RA on neuroblastoma were assessed. We demonstrated that treatment of SH-SY5Y cells with the combination of PtNPs and RA resulted in improved anticancer effects. The anticancer effects of the two compounds were mediated by cytotoxicity, oxidative stress (OS), mitochondrial dysfunction, endoplasmic reticulum stress (ERS), and apoptosis-associated networks. Cytotoxicity was confirmed by leakage of lactate dehydrogenase (LDH) and intracellular protease, and oxidative stress increased the level of reactive oxygen species (ROS), 4-hydroxynonenal (HNE), malondialdehyde (MDA), and nitric oxide (NO), and protein carbonyl content (PCC). The combination of PtNPs and RA caused mitochondrial dysfunction by decreasing the mitochondrial membrane potential (MMP), adenosine triphosphate (ATP) content, number of mitochondria, and expression of peroxisome proliferator-activated receptor gamma coactivator 1-alpha (PGC-1α). Endoplasmic reticulum-mediated stress and apoptosis were confirmed by upregulation of protein kinase RNA-like endoplasmic reticulum kinase (PERK), inositol-requiring enzyme 1 (IRE1), activating transcription factor 6 (ATF6), activating transcription factor 4 (ATF4), p53, Bax, and caspase-3 and down regulation of B-cell lymphoma 2 (BCl-2). PtNPs and RA induced apoptosis, and oxidative DNA damage was evident by the accumulation of 8-hydroxy-2-deoxyguanosine (8-OHdG) and 8-hydroxyguanosine (8-OHG). Finally, PtNPs and RA increased the differentiation and expression of differentiation markers. Differentiated SH-SY5Y cells pre-treated with PtNPs or RA or the combination of both were more sensitive to the cytotoxic effect of cisplatin than undifferentiated cells. To our knowledge, this is the first study to demonstrate the effect of the combination of PtNPs and RA in neuroblastoma cells. PtNPs may be a potential preconditioning or adjuvant compound in chemotherapeutic treatment. The results of this study provide a rationale for clinical evaluation of the combination of PtNPs and RA for the treatment of children suffering from high-risk neuroblastoma.

## 1. Introduction

Brain and central nervous system (CNS) cancers are associated with substantial morbidity and mortality worldwide. Primary brain and CNS cancers affect both children and adults, with more than 90% of the damage occurring in the brain and the remainder occurring in the meninges, spinal cord, and cranial nerves. The damage induced in brain and CNS cancers can ultimately lead to death [1,2]. The most common type of CNS cancer is glioma. In 2016, there were 330,000 cases of CNS cancer and 227,000 deaths globally, and age-standardized incidence rates of CNS cancer increased globally by 17.3% between 1990 and 2016. The highest incidence of CNS cancer for both sexes is in East Asia followed by Western Europe and South Asia. China, USA, and India have the highest number of cases of CNS cancer, and exposure to radiation is more prone to risk factors for CNS cancer is exposure to radiation.

The unique features of metallic nanoparticles can be explored in cancer therapy for diagnosis and surgery. For instance, irradiated gold nanoshells (AuNSs) and gold nanospheres have the potential to reduce the size of tumors in living animals [3,4,5,6]. Recently, a lot of effort has been directed towards the development of NIR resonant plasmonic nanoparticles with sizes of approximately 50 nm for systemic administration due to enhanced permeability and retention effect [7,8]. Although several nanogold materials have been used as NIR resonant nanoparticles, these particles have some limitations. Therefore, alternative plasmonic nanoparticles such as platinum nanoparticles (PtNPs) are very suitable for medical applications because of their thermal stability, photothermal efficiency, optical trapping, and enhanced spectroscopic signals [9,10,11]. Therefore, PtNPs are promising alternative candidates for NIR-based bioengineering purposes and thermoplasmonic applications [10].

Currently, cancer therapy is mostly based on the combinations of cytotoxic drugs with other therapeutic agents shows attractive and effective alternative therapeutic agents [12,13,14]. The combination of two or more therapeutic agents or strategies presents unique advantages, including the inhibition of different signaling pathways, target-specific actions, improved drug efficacy, and reduced off-target toxicity [15,16]. Retinoic acid (RA) plays important roles in cell development, differentiation, and cancer treatment particularly neuroblastoma, which is a solid tumor that occurs in children [17,18,19]. Currently, neuroblastoma is treated with a differentiating agent, RA, at the completion of cytotoxic therapy. Although RA improves survival rate by 35% in children, the 5-year event-free survival rate remains below 50% [20,21]. However, reversion has remained common, suggesting that minimal residual disease is an important cause of recurrence [22]. It has been reported that all-trans retinoic acid (ATRA) inhibits proliferation and induces differentiation in leukemia and glioblastoma cells [23]. The combination of ATRA and cisplatin exhibited enhanced cytotoxicity against squamous, head and neck, and ovarian cancer cells [24]. Similarly, in animal models, other retinoids are also involved in growth inhibition, differentiation, and proapoptotic effects in tumors of various organs, including the mouth, skin, bladder, lung, prostate, and breast [25]. ATRA regulates the phosphorylation and inactivation of the antiapoptotic protein Bcl-2 [26]. RA suppresses tumor growth by inducing cell differentiation, inhibiting cell proliferation, and exerting anti-migration and invasion effects on tumor cells [27]. ATRA suppressed the growth of human breast cancer cells (MCF-7) and hepatocellular carcinoma [28]. Several studies have reported the synergistic therapeutic effects of ATRA and its derivatives in combination with various anticancer drugs, including doxorubicin, CDDP, and paclitaxel, which were facilitated by the induction of receptor-mediated cytotoxicity and inhibition of cell growth factors [29,30,31]. In addition, it has been reported that ATRA (tretinoin) and 13-*cis*-retinoic acid (isotretinoin) decreased proliferation and induced differentiation in neuroblastoma cells [32]. Therefore, the use of RA in combination with chemotherapeutic agents enhances the therapeutic efficacy with reduced side effects.

Combination therapy is more attractive, more efficient, and has fewer side effects than single chemotherapy. Particularly, the combination of chemotherapeutic agents with biologically synthesized nanoparticles potentially induces apoptosis in various types of cancer cells [33,34]. The combination of retinoic acid and histone deacetylase inhibitors exhibited enhanced inhibitory effect against the growth of human neuroblastoma SH-SY5Y cells [35]. Similarly, the combination of HDAC inhibitor, LAQ 824, and 13-*cis*-retinoic acid exhibited stronger anticancer activity against melanoma tumors [36]. Intracranial tumors in ND2:SmoA1 mice treated with retinoid acid, SAHA, and cisplatin exhibited enhanced caspase-dependent apoptosis through the activation of caspase 3, reduced colony formation, cell migration in vitro, and tumorigenicity in vivo [37]. ATRA regulates the progression of cell cycle and increases the sensitivity of cells to anti-cancer agents in some type of cancers [29,38]. ATRA can reduce the proliferation of MCF-7 breast carcinoma cells and induce apoptosis, and its combination with omega-3 fatty acids can significantly reduce the progression of cancer cells. Nanotechnology-based combination therapy has significant advantages against cancer due to the stability and the ability to prolong drug exposure in blood circulation, improve pharmacokinetic parameters, and enhance tumor accumulation and cellular uptake of drugs [16,39,40,41,42]. Nanomedicine improves the efficiency of cancer treatment and overcomes limitations such as drug resistance, off target effects, and metastasis through targeted and multimode efficacy [43,44,45,46]. It is imperative to develop effective therapeutic strategies against cancer to improve long-term survival of patients. Therefore, effective non-surgical strategies and identification of the associated molecular mechanisms are crucial for the control of growth and apoptosis in neuroblastoma. In this study, we determined, for the first time, the effects of the combination of PtNPs and RA against neuroblastoma cancer cells, with regard to cytotoxicity, oxidative stress, mitochondrial dysfunction, endoplasmic reticulum stress (ERS), apoptosis, and differentiation.

## 2. Results and Discussion

### 2.1. Synthesis and Characterization of PtNPs Using Beta Carotene

**Figure 1:**
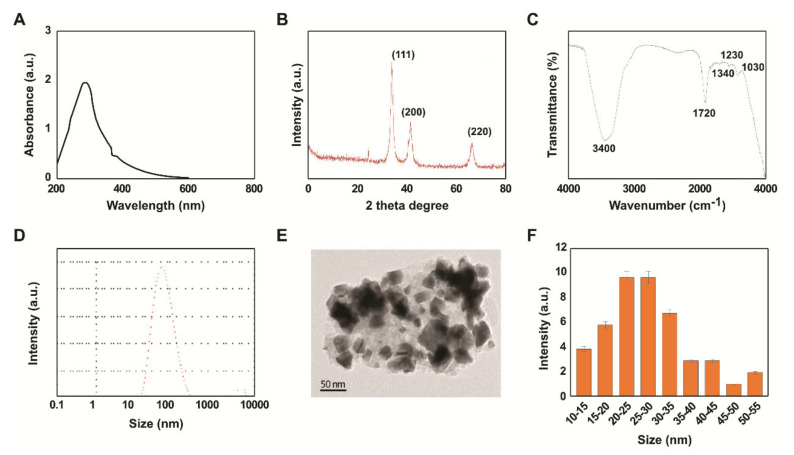
Synthesis and characterization of platinum nanoparticles (PtNPs) using β-carotene. Absorption spectra of beta carotene-mediated synthesis of PtNPs (**A**). X-ray diffraction patterns of PtNPs (**B**). FTIR spectra of PtNPs (**C**). Size distribution analysis of PtNPs using DLS (**D**). TEM images of PtNPs (**E**). Histograms showing particle size distribution (**F**). At least three independent experiments were performed for each sample and reproducible results were obtained. The data represent the results of a representative experiment. PtNPs, platinum nanoparticles; FTIR, Fourier-transform infrared; DLS, dynamic light scattering; TEM, transmission electron microscopy.

We developed an approach to synthesize PtNPs using beta carotene as templates. In a typical synthesis, 10 mL of 1 mg/mL beta carotene was added to 90 mL of 1 mM H_2_PtCl_6_. 6H_2_O solution and stirred for 30 min at 100 °C. During the reaction, a dark brownish color was formed, which suggested the formation of PtNPs. Further characterization of PtNPs was performed by ultraviolet-visible spectroscopy. PtNPs showed a typical characteristic peak at 290 nm, which confirmed the complete reduction of Pt (IV) ions to PtNPs (Figure 1A). In this study, the synthesis of PtNPs using a biological template was non-toxic, environmentally friendly, and non-hazardous, which was consistent with the reports of previous studies that had used various biomolecules such as *Quercus glauca* extracts, apigenin, tangeretin, and Saudi’s date extract, to synthesize PtNPs [47,48,49,50].

Next, we examined the crystalline structure and phase purity of as-prepared PtNPs with X-ray diffraction (XRD) analysis. Figure 1B depicts the XRD pattern of PtNPs synthesized using beta carotene. The three distinct and sharp diffraction peaks in the two theta degree range at 39.4, 45.7, and 66.1, correspond to (111), (200), and (220), respectively. The crystallographic plane of platinum is face-centered cubic (fcc) (JCPDS #87-0644). There were no other impurities, and conspicuous peaks were detected, demonstrating that as-prepared PtNPs had a highly crystalline nature [48,49,50]. Beta carotene-mediated reduction of Pt (IV) ions to PtNPs was further confirmed by Fourier-transform infrared (FTIR) spectroscopy. As shown in Figure 1C, FTIR spectra of PtNPs show the bands at 3400, 1720, 1340, 1230, and 1030 cm^−1^. The broad bands at around 3100–3500 cm^−1^ are attributed to the -OH groups of phenolic compounds (flavonoids), tannins, and –NH stretching of proteins [48,49,50]. The peaks at 1720 cm^−1^ represent the C=O stretching of carboxylic acids. The bands at 1340 cm^−1^ correspond to the C-H bending vibrations of CH_2_, whereas the bands at 1230 and 1030 cm^−1^ correspond to C-N stretching of aliphatic amine and C-O-C stretching of ether or glycoside groups, respectively [51]. The results from this study suggest that functional groups such as flavonoids, tannins, carboxyl, amino, and glycosides or ether groups are mainly responsible for the reduction and stabilization of Pt ions to PtNPs.

Furthermore, we determined the size of the nanoparticles, which is an essential parameter in the toxicity analysis of any prepared nanoparticles, by using dynamic light scattering. The results showed that the average particle size was 20–110 nm (Figure 1D). Using the dried powder of PtNPs, we further confirmed the size, shape, and morphology of PtNPs with transmission electron microscopy (TEM). The TEM micrograph image provided the shape, size, and morphology of the synthesized PtNPs. Interestingly, the particles showed various morphologies such as spherical, triangle, cubic, oval, hexagonal, and rod shapes (Figure 1E). The histogram from TEM image clearly indicates that the size of the PtNPs is between 10 and 50 nm with an average of 25 nm (Figure 1F), which is smaller than that determined by TEM. Nanoparticles with average size between 25 and 40 nm can penetrate and be internalized into tissues and cells very easily and rapidly, regardless of the core composition or surface charge of the nanoparticles [52]. The variety of shapes of nanoparticles plays critical roles in nanomedicine, including in therapeutic delivery processes such as particle adhesion, distribution, and cell internalization.

### 2.2. Effect of PtNPs and RA on the Viability of Various Types of Cancer Cells

**Figure 2:**
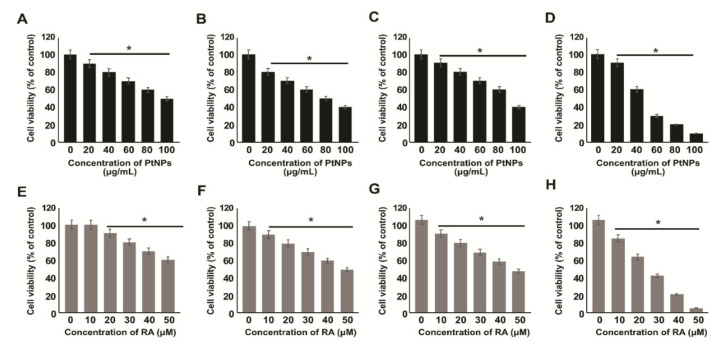
Determination of sensitivity of various type of cancer cells to PtNPs and retinoic acid (RA). Cell viability was determined in various types of cancer cells, including human adenocarcinoma cells (**A**,**E**), human breast cancer cells (**B**,**F**), human prostate cancer cells (**C**,**G**), and human neuroblastoma cells (**D**,**H**) treated with various concentrations of PtNPs (20–100 µg/mL) (**A**–**D**) or RA (10–50 µM) (**E**–**H**) for 24 h. The results are expressed as mean ± standard deviation of three independent experiments. The treated groups showed statistically significant differences from the control group per the Student’s *t*-test; * *p* < 0.05 was considered significant, PtNPs, platinum nanoparticles; RA, retinoic acid.

To identify the synergistic efficacy of PtNPs and RA and their sensitivity to cancer cells, we selected four different types of cancer cells, which included human adenocarcinoma cells (A549), human breast cancer cells (MDA-MB-231), human prostate cancer cells (LNCaP), and human neuroblastoma cancer cells (SH-SY5Y). Next, we examined the effects of PtNPs and RA on cell viability by treating the cells with various concentrations of PtNPs (0, 20, 40, 60, 80, and 100 μg/mL) or RA (0, 10, 20, 30, 40, and 50 μM) for 24 h. A dose-dependent loss of cell viability was observed in the cancer cells treated with PtNPs or RA for 24 h, whereas there was no significant difference in the viability of untreated cells. The results suggest that all the cancer cells showed similar trend of loss of cell viability with PtNP (Figure 2A–D) and RA (Figure 2E–H) treatment. However, SH-SY5Y cells showed more sensitivity against PtNPs and RA, followed by LNCaP, MD-MBA-231, and A549 cells. Therefore, to study the effect of the combination of PtNPs and RA, we determined the IC_50_ and IC_25_ as well as the dose-dependent effects of PtNPs, RA, PtNPs, and RA, and cisplatin on the viability of SH-SY5Y cells.

### 2.3. Dose-Dependent Effects of PtNPs, RA, and Cisplatin on the Viability and Proliferation of SH-SY5Y Cells

**Figure 3:**
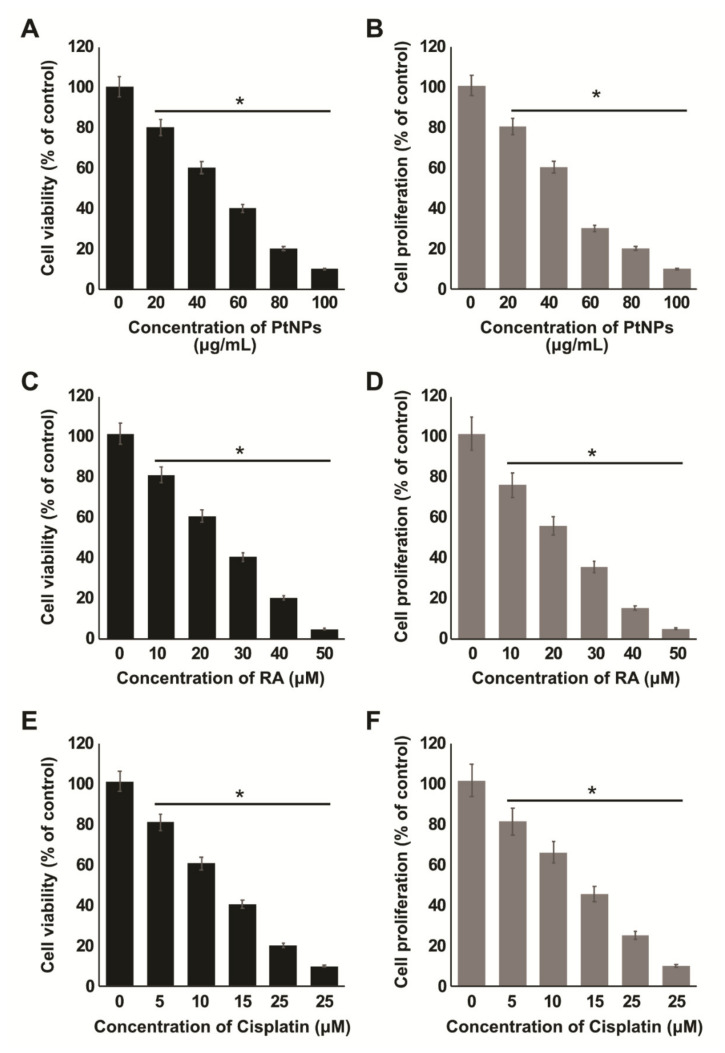
Dose-dependent effect of PtNPs, RA, and cisplatin on cell viability and proliferation of SH-SY5Y cells. The viability of SH-SY5Y cells was determined after 24-h exposure to different concentrations of PtNPs (20–100 µg/mL) (**A**). Proliferation of SH-SY5Y cells was determined using the BrdU assay after 24-h exposure to different concentrations of PtNPs (20–100 µg/mL) (**B**). Viability of SH-SY5Y cells was determined after 24-h exposure to different concentrations of RA (10–50 µM) (**C**). Proliferation of SH-SY5Y cells was determined using the BrdU assay after 24-h exposure to different concentrations of PtNPs (10–50 µM) (**D**). Viability of SH-SY5Y cells was determined after 24-h exposure to different concentrations of cisplatin (5–25 µM) (**E**). Proliferation of SH-SY5Y cells was determined using the BrdU assay after 24-h exposure to different concentrations of cisplatin (5–25 µg/mL) (**F**). The results are expressed as mean ± standard deviation of three independent experiments. The treated groups showed statistically significant differences from the control group per the Student’s *t*-test; * *p* < 0.05 was considered significant. PtNPs, platinum nanoparticles; RA, retinoic acid.

In order to determine the efficacy of the combination of PtNPs and RA, we first determined the dose-dependent effects of PtNPs, RA, and cisplatin individually on the viability and proliferation of the treated cells. SH-SY5Y cells were treated with various concentrations of PtNPs (10–100 µg/mL) for 24 h. We observed significant concentration-dependent cytotoxicity after treatment with PtNPs for 24 h (Figure 3A). Similarly, the cells treated with PtNPs showed significantly lower proliferation than normal cells (Figure 3B). We found that the IC_25_ and IC_50_ values of PtNPs against SH-SY5Y cells were 25 ± 1.50 and 50 ± 1.70 µg/mL, respectively. With an increase in the concentration of PtNPs, we observed an increase in cell death and a decrease in the rate of proliferation of cancer cells, which confirms that the concentration of PtNPs has a significant role in controlling the number of cancer cells. Based on these results, we selected 25 µg/mL as the concentration of PtNPs to be used in combination with RA. Platinum-based materials show significant toxicity towards cancer cells. For instance, Pt quantum dots showed dose- and time-dependent cytotoxic effects in C2C12 myoblast cancer cells [52]. Bendale et al. [53] reported the cytotoxic effects of ptNPs in various human lung adenocarcinoma (A549), ovarian teratocarcinoma (PA-1), pancreatic cancer (Mia-Pa-Ca-2) cells, and normal peripheral blood mononucleocyte (PBMC) cells. U87 glioma cells treated with PtNPs exhibited altered morphology, loss of viability, increased mortality rate, and genotoxicity [54]. Almeer et al. [55] reported the dose- and time-dependent toxicity of biologically prepared PtNPs in HEK293 cells, using various concentrations from 20 to 360 µg/mL, noting that significant toxicity occurred at higher concentrations. In this study, our findings revealed that the effect of 25.0 µg/mL PtNPs was significant, which may be attributed to the size of the particles. Graphene oxide-platinum nanoparticle nanocomposites showed significant toxicity through increased cell death and reduced cell proliferation in prostate cancer cells [56]. Apigenin-functionalized ultra-small PtNPs induced cytotoxicity in a dose-dependent manner by reducing cell viability and proliferation in human monocytic THP-1 cells [48]. Similarly, zinc oxide nanoparticles induced cell death in dose- and time-dependent manners in SH-SY5Y cells [57], and tangeretin-assisted PtNPs showed dose-dependent cytotoxicity against human bone OS epithelial cells [49]. Altogether, beta carotene-functionalized PtNPs have significant cytotoxic effects against human neuroblastoma cells, which are mediated by the reduction in cell viability and cell proliferation.

Next, we evaluated the effects of RA on cell growth in SH-SY5Y cells. As shown in Figure 3C, cell viability was reduced, as detected with the cell counting kit-8 assay, in a dose-dependent manner. At 24 h, we found that the IC_25_ and IC_50_ values of RA against SH-SY5Y cells were 12.5 ± 1.00 and 25. 0 ± 1.60 μM, respectively, which indicates a possibility that the SH-SY5Y cells were more sensitive to the inhibitory effects of RA. We further determined the effect of RA on cell proliferation and observed that RA exhibited a strong inhibitory effect on the proliferation of SH-SY5Y cells (Figure 3D). In fact, 25 µM RA caused 50% reduction in cell viability and proliferation, which indicates that RA inhibition of SH-SY5Y cell growth is concentration-dependent. For each group, the difference was statistically significant (*p* < 0.05), in comparison with the control group. These results were consistent with the report of a study where ATRA inhibited cell growth and caused cell death dose-dependently in different type of breast cancer cells such as 4T1, EMT6, and MDA-MB-231 cells [58].

Cisplatin is an effective drug for the treatment of neuroblastoma and other cancers, and hence, we selected this drug as a positive control for all the experiments. In addition, to determine the effect of cisplatin on PtNPs and RA-differentiated cells, we determined the inhibitory effect of cisplatin on the viability and proliferation of SH-SY5Y cells. The cells were exposed to various concentrations of cisplatin, and cell viability and proliferation were determined using the CCK-8 and BrdU assays, respectively. We found that the IC_25_ and IC_50_ values of cisplatin against SH-SY5Y cells were 6.5 ± 1.00 and 12. 5. ± 1.20 μM, respectively. The cell viability and proliferation rate of cisplatin-treated cultures were inhibited by ~50% using a concentration of 12.50 ± 1.20 μM cisplatin (Figure 3E). The number of proliferating cells significantly decreased with increasing concentrations of cisplatin, as observed from the results of the cell viability and BrdU assays (Figure 3F). The percentages of cell death also increased with increasing concentrations of cisplatin. These results indicate that cisplatin inhibited the viability and proliferation of SH-SY5Y cells in a dose-dependent manner, which is consistent with the report of Sun et al. [59].

### 2.4. Effect of the Combination of PtNPs and RA on the Viability, Proliferation, and Morphology of SH-SY5Y Cells

**Figure 4:**
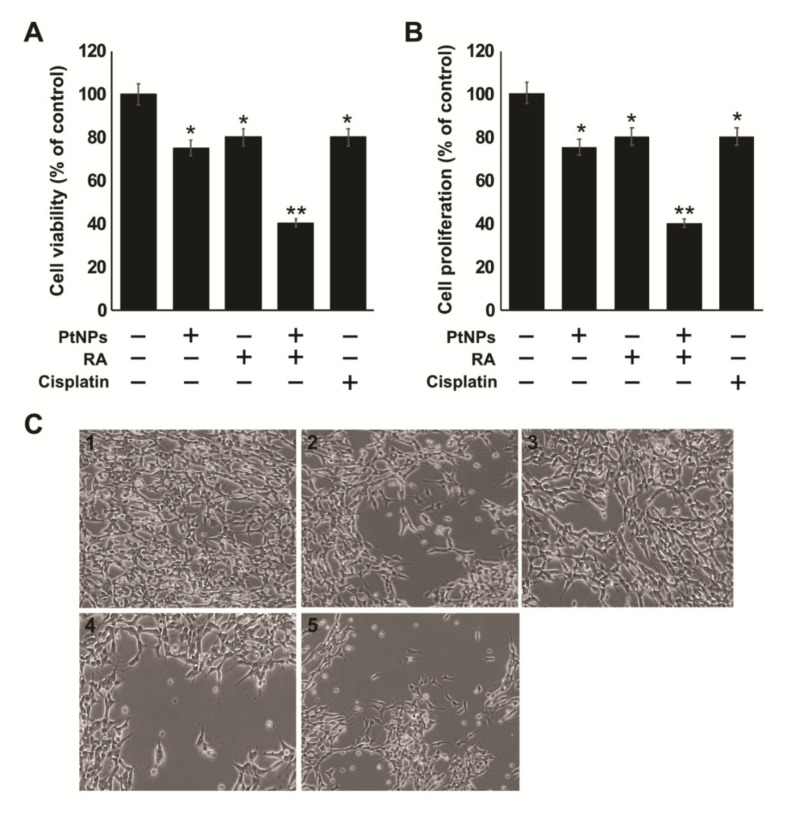
Combination of PtNPs and RA induces loss of cell viability, reduces proliferation, and alters cell morphology in SH-SY5Y cells. SH-SY5Y cells were incubated with PtNPs (25 μg/mL), RA (12.5 μM), combination of PtNPs (25 μg/mL) and RA (12.5 μM), or cisplatin (6.5 μM) for 24 h. Cell viability (**A**), cell proliferation (**B**), were determined. The results are expressed as mean ± standard deviation of three independent experiments. The treated groups showed statistically significant differences from the control group per the Student’s *t*-test; * *p* < 0.05 was considered significant, ** *p* < 0.01 was considered highly significant. PtNPs, platinum nanoparticles; RA, retinoic acid. Cell morphology control (1), PtNPs (2), RA (3), combination of PtNPs and RA (4), or cisplatin (5) for 24 h (**C**). Scale bar-200 µm

The selection of dose is very crucial for combination studies. After establishing the IC_25_ and IC_50_ of PtNPs and RA, the effects of their combination were tested. The IC_25_ values of PtNPs, RA, and cisplatin were used in all the experiments, which were 25 μg/mL, 12.5 μM, and 6.5 μM, respectively, unless otherwise specified. The present study aimed to elucidate the combined effects of PtNPs and RA on the suppression of viability and proliferation of SH-SY5Y cells. We selected IC_25_ for PtNPs (25 µg/mL) and RA (12.5 µM) to avoid high lethality to the cells and reduce drug resistance in cancer cells by chemosensitization through additive or synergistic effects. The cells were treated with PtNPs (25 µg/mL) and RA (12.5 µM) or cisplatin as positive control for 24 h. Cisplatin exhibited moderate effect, in comparison with the combination of PtNPs and RA. As depicted in Figure 4A,B, the cytotoxic effect of PtNPs in combination with RA was enhanced, compared with that of PtNPs alone. The results revealed that treatment with the combination of PtNPs and RA had an enhanced effect on SH-SY5Y cells compared with that of control, and the differences were found to be significant (*p* < 0.01), indicating synergistic effects. It has been reported that the combination of RA and histone deacetylase inhibitors potentially inhibited the growth of neuroblastoma SH-SY5Y cells, compared with the individual compounds [35]. Similarly, Hong et al. [60] reported that the combination of paclitaxel and ATRA (10 + 10 μg/mL) delivered by nanoparticles showed a synergistic antiproliferative effect against CT26 cells. Zhu et al. [58] reported that pluronic-ATRA synergistically enhanced the cytotoxic effects of cisplatin and effectively suppressed breast tumor growth in vivo.

Morphological changes are the hall mark of cell death. Using morphological analysis, cell death was determined in SH-SY5Y cells treated with PtNPs, RA, or PtNPs and RA. SH-SY5Y cells were treated with PtNPs, RA, or PtNPs and RA for 24 h, and the changes in cell morphology were observed after 24 h. Bright field images clearly demonstrated that treated SH-SY5Y cells displayed morphological alterations typically associated with apoptotic cell death. All the treated cells showed cell shrinkage, membrane bleb formation, aggregation, and detachment from the cell surface (Figure 4C). Morphological changes became visible, and numerous vacuoles were observed in the cytosol. Cell morphology was markedly altered, an effect that was clearly similar to that of the apoptosis-inducing agent, cisplatin, which suggested that a combination of PtNPs and RA induced cell death in the cancer cells. The results suggest that PtNPs and RA reduced cell proliferation and induced marked changes in cell morphology, including the appearance of long cytoplasmic protrusions and a general neuron-like phenotype. Cells treated with the combination of PtNPs and RA were morphologically distinct from untreated cells. Santos et al. [35] observed that SH-SY5Y cells treated with RA exhibited morphologic differentiation with neurite extension. However, the combination of HDACi, SAHA, and RA induced cell death, with neurite shortening. The combination of PtNPs and RA significantly compromised cellular integrity and led to more cell death, compared with either PtNPs or RA individually. In a similar study, treatment with the combination of ATRA and LOX/COX inhibitors increased cell death by inhibiting cell proliferation and inducing noticeable changes in cell morphology, which comprised long cytoplasmic protrusions and a general neuron-like phenotype [61].

### 2.5. Combination of PtNPs and RA Induces Lactate Dehydrogenase (LDH) Leakage and Intracellular Protease

**Figure 5:**
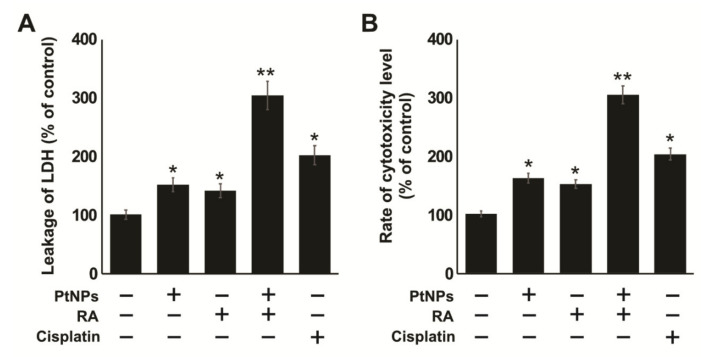
Combination of PtNPs and RA induces cytotoxicity in SH-SY5Y cells. SH-SY5Y cells were incubated with PtNPs (25 μg/mL), RA (12.5 μM), combination of PtNPs (25 μg/mL) and RA (12.5 μM), or cisplatin (6.5 μM) for 24 h. Leakage of lactate dehydrogenase (LDH) was measured at 490 nm using the LDH cytotoxicity kit (**A**), and the level of dead-cell protease was determined by CytoTox-Glo cytotoxicity assay (**B**). The results are expressed as mean ± standard deviation of three independent experiments. The treated groups showed statistically significant differences from the control group per the Student’s *t*-test; * *p* < 0.05 was considered significant, ** *p* < 0.01 was considered highly significant. PtNPs, platinum nanoparticles; RA, retinoic acid.

Cells treated with cytotoxic compounds, such as PtNPs, are killed through swelling and the loss of membrane integrity, after which they shut down and release their intracellular contents into the surrounding environment. When the cell membranes are compromised, lactate dehydrogenase (LDH), a soluble, stable enzyme and a typical marker of cell death, is released into the surrounding extracellular space. To determine the effect of PtNP and RA combination, SH-SY5Y cells were treated with PtNPs, RA, or PtNPs and RA for 24 h, and the LDH level was measured. The results showed that the effect of PtNP and RA combination was significantly stronger than that of PtNPs or RA, and LDH level was 3-fold higher than that with individual treatment (Figure 5A). Cisplatin showed moderate effect compared with the control. These findings clearly suggest that the cell membrane integrity was severely compromised by PtNPs and RA. For example, when T47D cells were exposed to tamoxifen and simvastatin combination, the leakage of LDH was significantly higher than that observed with the control treatment [62]. We have previously reported that treatment with the combination of silver nanoparticles and histone deacetylase inhibitors significantly increases the leakage of LDH, compared with that on individual treatment of human alveolar basal epithelial cells [63]. Collectively, the release of LDH suggests that necrosis as well as necroptosis could be involved in PtNP- and RA-induced SH-SY5Y cell death.

Determination of intracellular protease is an alternative method for the determination of cell viability and cytotoxicity. Measurement of protease level provides quantitative data on the number of live or dead cells in a sample. SH-SY5Y cells were treated with PtNPs, RA, or PtNPs and RA for 24 h, and the intracellular protease was measured. The results showed that the combined effect of PtNPs and RA was significantly higher, and the protease level was 3-fold higher than that on treatment with PtNPs or RA (Figure 5B). However, PtNPs, RA, and cisplatin showed moderate effect compared with the control. The findings of this study clearly suggest that the integrity of the cell membrane was severely compromised by PtNPs and RA. In a previous study, the combination of silver nanoparticles and gemcitabine had shown enhanced cytotoxicity potential against human ovarian cancer cells by increasing the level of protease [64]. Similarly, the combination of silver nanoparticles and HDAC inhibitor increased the leakage of protease in human alveolar basal epithelial cells [63]. In addition, the combination of graphene oxide-silver nanoparticles and trichostatin A enhanced the leakage of intracellular protease in human ovarian cancer cells [65], and RA increased the leakage of LDH in F9 teratocarcinoma stem cells [66]. In this study, the rate of intracellular protease leakage into the cell medium was directly proportional to the cytotoxicity of PtNPs and RA against SH-SY5Y cells.

### 2.6. PtNPs and RA Enhance the Production of Reactive Oxygen Species (ROS), 4-hydroxynonenal (4-HNE), Malondialdehyde (MDA), Nitric Oxide (NO), and Protein Carbonyl Content (PCC)

**Figure 6:**
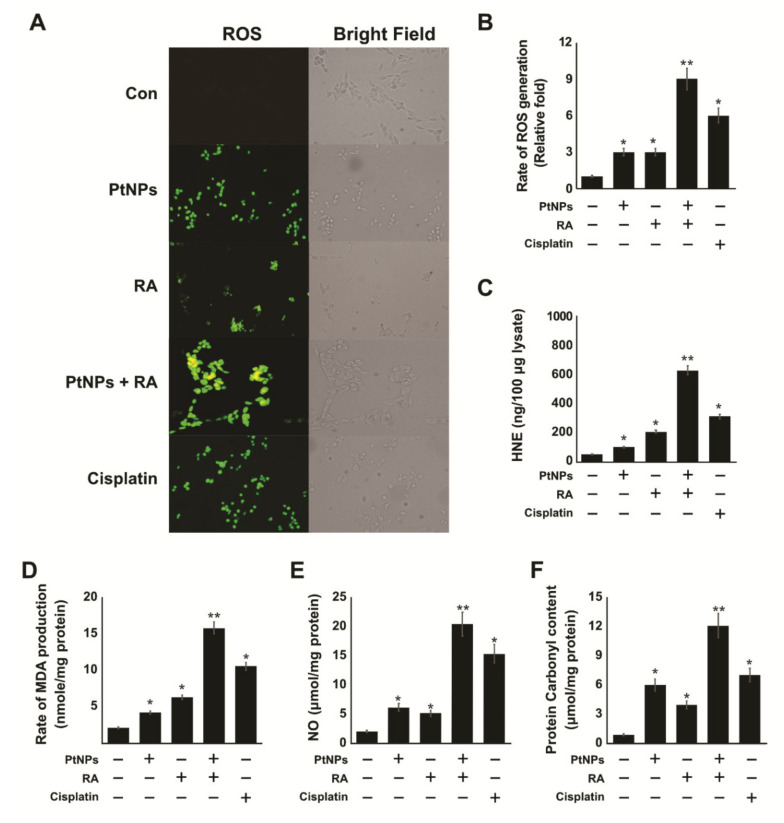
PtNPs and RA increase the generation of ROS, lipid peroxidation, nitric oxide (NO) level, and protein carbonylation. SH-SY5Y cells were incubated with PtNPs (25 μg/mL), RA (12.5 μM), combination of PtNPs (25 μg/mL) and RA (12.5 μM), or cisplatin (6.5 μM) for 24 h. Treated SH-SY5Y cells were subjected to 2′,7′-dichlorodihydrofluorescein diacetate-fluorescein isothiocyanate (DCFH-DA-FITC) analysis (**A**) (Scale bar-100 µm). Spectrophotometric analysis of ROS using 2′,7′-dichlorodihydrofluorescein diacetate (DCFH-DA) (**B**). 4-HNE level was determined using enzyme-linked immunosorbent assay (ELISA) (**C**). MDA concentration was measured using a thiobarbituric acid-reactive substances assay and was expressed as nanomoles per gram of protein (**D**). NO production was quantified spectrophotometrically using the Griess reagent and expressed as µM per g of protein (**E**). Protein carbonylation content was determined and expressed as µM per g of protein (**F**). The results are expressed as mean ± standard deviation of three independent experiments. The treated groups showed statistically significant differences from the control group per the Student’s *t*-test; * *p* < 0.05 was considered significant, ** *p* < 0.01 was considered highly significant. PtNPs, platinum nanoparticles; RA, retinoic acid; DCFH-DA-FITC, dichloro-dihydro-fluorescein diacetate; 4-HNE, 4-hydroxynonenal; ELISA, enzyme-linked immunosorbent assay; MDA, malondialdehyde.

Mitochondria generated ROS can induce an intracellular state of oxidative stress, leading to permanent cell damage. Thus, the intracellular accumulation of ROS not only disrupts the functions of organelles but also leads to cytotoxicity. Fluorescence microscopy was used to visualize ROS staining and the involvement of mitochondrial oxidative stress. To determine the level of ROS induced by treatment with PtNPs and RA, we used a fluorescent probe, dichloro-dihydro-fluorescein diacetate (DCFH-DA), to investigate the extent of oxidative stress. The fluorescence intensity of DCF can directly reflect the level of reactive oxygen species (ROS) in the cell. SH-SY5Y cells were treated with PtNPs, RA, or PtNPs and RA for 24 h. The cells were stained with DCFH-DA, and the production of ROS was determined by measuring the cell population that was positive for DCF-derived fluorescence after the treatment. Treatment with PtNPs and RA induced ROS generation as shown in Figure 6A. The intensity of ROS signal generated in the cells treated with PtNPs and RA was significantly higher than that in the control (*p* < 0.001), and the bright green fluorescence was distributed in a granular manner within the cytoplasm. The intensity of ROS in the cells treated with PtNPs, RA, and cisplatin was higher than that in the untreated cells and lower than that in the cells treated with the combination of PtNPs and RA. To further confirm the generation of ROS, we measured ROS by spectrophotometric analysis after the cells were exposed to PtNPs and RA for 24 h, and the amount of intracellular ROS detected was 3-fold higher than that in the PtNPs- or RA-treated group and 9-fold higher than that in the control group (Figure 6B). This indicates that PtNPs accelerated the production of intracellular ROS. Interestingly, the results demonstrated a significant difference in the production of ROS in cells treated with PtNPs and RA, compared with those treated with either PtNPs or RA. Both PtNPs and RA have been shown to exhibit cytotoxic and anticancer activities through the induction of oxidative stress by generating ROS in various types of cells including human embryonic kidney cells [55], A549 lung carcinoma cells [67], human monocytic THP-1 cells [48], and OS epithelial cells [49].

Recently, different studies have focused on lipid peroxidation (LPO) products and intracellular signaling mechanisms that determine the fate of cells [68,69]. Generally, LPO arises from the oxidation of fatty acids induced by oxidants. However, there is little evidence that nanoparticles induce the production of LPO. In this study, we were interested in determining the effect of the combination of PtNPs and RA on the levels of 4-hydroxynonenal (4-HNE), malondialdehyde (MDA), nitric oxide (NO), and protein carbonyl content (PCC). Oxidative stress is a common feature of NPs, and to examine the hypothesis that oxidative stress mediates the induction of the accumulation of 4-HNE, SH-SY5Y cells were treated with PtNPs, RA, or PtNPs and RA for 24 h, and the level of 4-HNE was measured using enzyme-linked immunosorbent assay (ELISA). The accumulation rate of 4-HNE (600 μg/100 μg lysate) in the group of cells treated with PtNPs and RA was significantly higher than that in cells treated with either PtNPs (100 μg/100 μg lysate) or RA (200 μg/100 μg lysate) (Figure 6C). The accumulation rate of 4-HNE in the cisplatin-treated group was 300 μg/100 μg lysate. Furthermore, another LPO marker, MDA, was used to confirm the lipid peroxidation state in the presence of PtNPs and RA. Results showed that the level of MDA was significantly higher (15 nM/mg of protein) with PtNP and RA treatment than with either PtNP (4 nM/mg of protein) or RA (4 nM/mg of protein) treatment (Figure 6D). In addition, the level of MDA in the cisplatin-treated cells was 10 nM/mg of protein. The production of LPO-derived aldehydes in cancer cells depends on the presence of ROS. The increased level of ROS can increase the formation of LPO products and eventually increase oxidative damage to DNA [70]. Exogenous application of 4-HNE inhibits cancer growth by inhibiting proliferation and inducing the apoptosis of cancer cells. 4-HNE inhibited the proliferation of human colon tumor cells, through regulation of the MAP kinases pathway [71], PPAR gamma pathway [72], and the activation of p53 in cancer cells [73].

NO is a key regulator of redox signaling and homeostasis, and reactive nitrogen species elicit various modifications of macromolecules to produce nitrative or nitro-OS. However, abnormal increased production of NO causes cellular damage [74]. Therefore, we investigated the impact of PtNPs, RA, and the combination of PtNPs and RA on nitro-OS-induced NO production. SH-SY5Y cells were treated with of PtNPs, RA, or PtNPs and RA for 24 h. We found that the combination of PtNPs and RA enhanced the production of NO in SH-SY5Y cells. As shown in Figure 6E, incubation of cells with PtNPs, RA, or PtNPs and RA for 24 h resulted in a significant increase in NO levels (*p* < 0.005). Several studies have suggested that NO induces neurotoxicity in neuronal cells via cytochrome *c* release into the cytosol and caspase activation in SH-SY5Y cells [75]. Increased level of NO causes mitochondrial damage via alteration of membrane potential and inhibition of mitochondrial respiratory chain [76,77]. Clementi et al. reported that NO created a sequence of events in the cells, which included ROS production, the loss of mitochondrial membrane potential, cytochrome *c* release into the cytosol, caspase activation, DNA fragmentation, and eventually apoptosis. Recent studies have suggested that PtNPs or RA induces NO production and ultimately causes cell death in various types of cancer cells, including SH-SY5Y cells [78], human monocytic THP-1 [48], and OS epithelial cells [49]. Altogether, these findings suggest that the combination of PtNPs and RA can potentially induce NO and that elevated NO could cause cell death in SH-SY5Y cells.

One of the crucial and important markers of oxidative stress is protein carbonylation, which is measured by estimation of the protein carbonyl groups. Protein carbonylation is a type of protein oxidation that can be promoted by ROS [79,80]. A significant increase (*p* < 0.005) in the levels of PCC was observed in TSH-SY5Y cells treated with PtNPs, RA, or the combination of PtNPs and RA for 24 h (6, 4, 12, and 7 μM/g of protein, respectively), compared with the untreated cells (Figure 6F). MRC-5 cells exposed to silica nanoparticles displayed loss of cell viability, which was associated with the induction of cellular oxidative stress, increased levels of carbonyl groups and advanced oxidation protein products, and the decreased concentration of glutathione (GSH) and protein sulfhydryl groups [81]. Similarly, the combination of PtNPs and doxorubicin enhanced protein oxidation in osteosarcoma cells [49]. Carbonylation is irreversible and ROS-induced protein oxidation causes loss of protein function, which is often associated with protein unfolding and aggregation as well as signal transduction [82,83]. It has also been reported that increased protein carbonylation causes various types of diseases such as metabolic diseases [84], neurodegenerative diseases [83], as well as aging and age-related diseases [85,86]. Collectively, all these findings suggest that oxidative stress is associated with the toxicity of PtNPs and RA in SH-SY5Y cells through increased levels of ROS, MDA, NO, and protein carbonylation.

### 2.7. PtNPs and RA Decrease Antioxidants Levels

**Figure 7:**
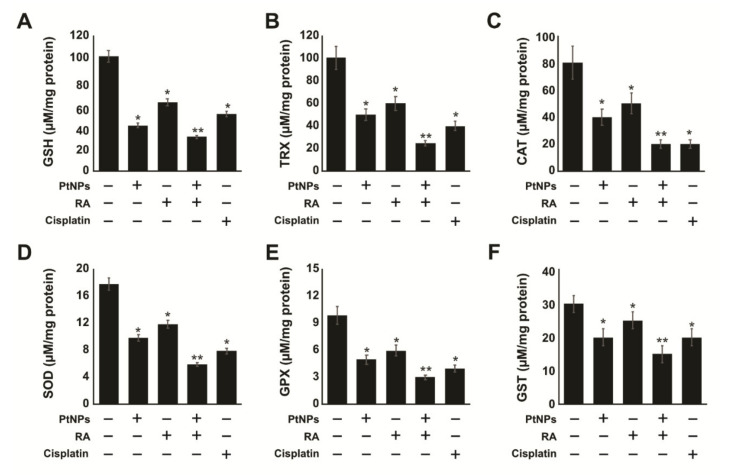
Effect of the combination of PtNPs and RA on antioxidant markers. SH-SY5Y cells were incubated with PtNPs (25 μg/mL), RA (12.5 μM), combination of PtNPs (25 μg/mL) and RA (12.5 μM), or cisplatin (6.5 μM) for 24 h. After incubation, the cells were harvested and washed twice with ice-cold phosphate-buffered saline solution. The cells were collected and disrupted by ultrasonication for 5 min on ice. Glutathione (GSH) concentration was expressed as µM per mg of protein (**A**). Thioredoxin (TRX) concentration was expressed as µM per mg of protein (**B**). Catalase (CAT) was expressed as unit per mg of protein (**C**). Superoxide dismutase (SOD) was expressed as unit per mg of protein (**D**). Glutathione peroxidase (GPx) concentration was expressed as µM per mg of protein (**E**). Glutathione S-transferase (GST) concentration was expressed as µM per mg of protein (**F**). Results are expressed as mean ± standard deviation of three independent experiments. There was a significant difference between treated and untreated cells per Student’s *t*-test (* *p* < 0.05). The treated groups showed statistically significant differences from the control group per the Student’s *t*-test; * *p* < 0.05 was considered significant, ** *p* < 0.01 was considered highly significant. PtNPs, platinum nanoparticles; RA, retinoic acid; GSH, glutathione; TRX, thioredoxin; CAT, catalase; SOD, superoxide dismutase; GPX, glutathione peroxidase; GST, glutathione-S-transferase.

Alteration of the balance of antioxidants/oxidants has a significant role in the pathogenesis of different kinds of tumors [87]. Enzymatic antioxidants play critical roles against oxidative stress. To evaluate the impact of PtNPs and RA on various antioxidant levels, SH-SY5Y cells were treated with PtNPs, RA, PtNPs and RA, or cisplatin for 24 h. All the tested antioxidants such as glutathione (GSH), thioredoxin (TRX), catalase (CAT), superoxide dismutase (SOD), glutathione peroxidase (GPx), and glutathione S-transferase (GST) were clearly downregulated in the treated cells, particularly, the combination of PtNPs and RA significantly and strongly downregulated the antioxidants by up to 3-fold compared with single PtNPs, RA, or cisplatin treatment. As shown in Figure 7, GSH levels in SH-SY5Y cells treated with PtNPs, RA, and cisplatin for 24 h were 40, 60, and 50 μM GSH, respectively, whereas exposure of the cells to the combination of PtNPs and RA for 24 h resulted in significant reduction in GSH level (30 μM). The same trend was observed for the TRX contents, which were 25, 50, 60, and 40 μM in the cells treated with PtNPs and RA, PtNPs, RA, and cisplatin for 24 h, respectively. In addition, antioxidants levels of CAT and SOD declined; the levels of CAT in SH-SY5Y cells exposed to the combination of PtNPs and RA, PtNPs, RA, and cisplatin for 24 h were 20, 40, 50, and 20 μM, respectively. Similarly, the SOD contents were 6, 10, 12, and 8 μM in cells treated with PtNPs and RA, PtNPs, RA, and cisplatin for 24 h, respectively (Figure 7).

TRX and GSH are the two major thiol-dependent antioxidants involved in the maintenance of cell homeostasis as well as DNA synthesis and repair [88,89]. Several animal model studies have demonstrated that the downregulated expression of TRX and GSH proteins causes aging and the overexpression of these two systems improves aging [90]. A previous study reported that phenethyl isothiocyanates killed malignant cancer cells by disabling GSH antioxidant system and disrupting redox-sensitive survival pathways [91]. Similarly, in this study, PtNPs and RA significantly decreased the levels of CAT and SOD, compared with PtNPs, RA, or cisplatin alone. We observed that decreasing CAT activity had a significant effect on the rate constant of H_2_O_2_ removal. Manganese superoxide dismutase (MnSOD) is involved in ROS homeostasis and exhibits tumor-suppressive and cancer-promoting functions [92]. ROS was reported to be majorly responsible for the downregulation of CAT via activation of PI3K/Akt signaling to reduce the activity of forkhead box protein O1 (FoxO1) in mesangial cells [93]. The reduced MnSOD activity in A431-P and A431-III cells promoted the metastatic ability of cancer cells [94]. As shown in Figure 7, the levels of GPx in SH-SY5Y cells exposed to PtNPs, RA, and cisplatin for 24 h were 5, 6, and 4 μM, respectively, whereas PtNP and RA treatment resulted in significant reduction in GPx level (3 μM). The same trend was observed for the GST contents, which were 15, 20, 25, and 20 μM for PtNPs and RA, PtNPs, RA, and cisplatin after 24 h of treatment, respectively. The significant decrease in the levels of all the antioxidants suggests that the antioxidative system adaptation was not enough to prevent damage induced by high-level oxidative stress produced by PtNPs and RA. This could affect the structure of DNA bases, proteins, and carbohydrates and eventually the proliferation of cancer cells. Like other metallic nanoparticles, PtNPs could decrease the levels of various antioxidants in various types of cancer cells, including SH-SY5Y cells, human monocytic THP-1, and OS epithelial cells. Collectively, our results indicate that increased production of ROS and decreased levels of antioxidants can initiate apoptosis in cancer cells.

### 2.8. PtNPs and RA Induce Mitochondrial Dysfunction

**Figure 8:**
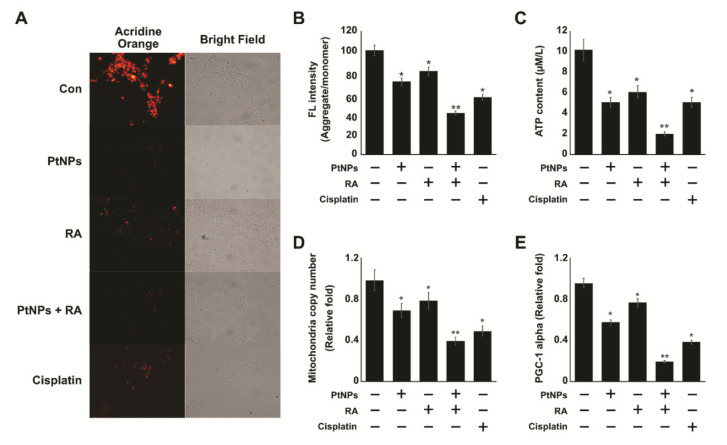
PtNPs and RA alter mitochondrial membrane potential (MMP), ATP content, mitochondrial copy number, and PGC1α expression. SH-SY5Y cells were incubated with PtNPs (25 μg/mL), RA (12.5 μM), combination of PtNPs (25 μg/mL) and RA (12.5 μM), or cisplatin (6.5 μM) for 24 h. Fluorescence microscopic images of the SH-SY5Y cells stained with JC-1 dye for detecting the changes in mitochondrial membrane potential (**A**) scale bar-100 µm. MMP was determined using the cationic fluorescent indicator JC-1 (**B**). SH-SY5Y cells were treated with PtNPs, RA, PtNPs and RA, or cisplatin for 24 h, and the intracellular ATP content was determined according to the manufacturer’s instructions (Sigma-Aldrich, St. Louis, MO, USA; Catalog Number MAK135) (**C**). Mitochondrial copy number (**D**) and the expression of PGC1α were determined by reverse transcription-quantitative polymerase chain reaction (RT-qPCR) analysis (**E**). The results are expressed as the mean ± standard deviation of three independent experiments. The treated groups showed statistically significant differences from the control group per the Student’s *t*-test; * *p* < 0.05 was considered significant, ** *p* < 0.01 was considered highly significant. PtNPs, platinum nanoparticles; RA, retinoic acid; RT-qPCR, reverse transcription-quantitative polymerase chain reaction; PGC1α, peroxisome proliferator-activated receptor gamma coactivator 1-alpha.

Mitochondria not only provide energy for the cell but are also a source of ROS. They regulate various other cellular functions, including calcium signaling, membrane potential regulation, cell proliferation, and apoptosis. The production of ROS is tightly regulated by the antioxidant system, and imbalance between prooxidants and antioxidants causes mitochondrial dysfunction. Therefore, we decided to measure mitochondrial membrane potential in SH-SY5Y cells exposed to PtNPs, RA, PtNPs and RA, and cisplatin for 24 h. Mitochondrial membrane potential (MMP) is a key indicator of membrane integrity, and changes in MMP lead to apoptosis. Therefore, the effect of PtNPs, RA, PtNPs and RA, and cisplatin on the MMP of SH-SY5Y cells was evaluated using fluorescence microscopy. As shown in Figure 8A, JC-1 dye exhibited potential-dependent accumulation of mitochondria, indicated by a fluorescence emission shift from green to red. In the cells treated with PtNPs, RA, PtNPs and RA, or cisplatin, mitochondrial depolarization occurred as demonstrated by the decreased red/green fluorescence intensity ratio. Moreover, the color shift from red to green or loss of red fluorescent J-aggregates indicated disruption caused by PtNPs, RA, PtNPs and RA, and cisplatin. Particularly, the cells treated with PtNPs and RA showed significant loss of membrane integrity, compared with the control. Furthermore, to gain insight into the effect of the combination of PtNPs and RA in SH-SY5Y cells, we examined fluorescence intensity by determining aggregate/monomer ratios, which were compared with the control value (100%). Treatment of SH-SY5Y cells with PtNPs and RA caused significant loss of MMP through the reduction of aggregates by 60 ± 4.0%, compared with the control. However, the cells treated with PtNPs, RA, or cisplatin lost MMP through the reduction of aggregates by 30, 20, and 45 ± 4.5%, respectively (Figure 8B). Significant difference was observed in all treated samples, compared with the control group. These results demonstrate that mitochondrial depolarization was induced by PtNPs and RA. Kusaczuk et al. reported that compared with the control cells, LN229 cells treated with SiNPs exhibited a significant decrease in ΔΨ_m_, as approximately 58% of the cells independently showed decreased ΔΨ_m_. PtNPs decreased the level of MMP in various types of cancer cells, including SH-SY5Y [78], human monocytic THP-1 [48], and OS epithelial cells [49]. In addition, to confirm mitochondrial toxicity, the rate of ATP synthesis was measured in the cells. The results showed that SH-SY5Y cells treated with the combination of PtNPs and RA showed significantly lower ATP generation than those treated singly with PtNPs, RA, or cisplatin (Figure 8C). Importantly, after 24 h of incubation with PtNPs and RA, ATP generation was significantly attenuated, compared with that in the single treatment groups or untreated group.

Mitochondria play significant roles in the metabolic activity of the cells, and impairment of mitochondrial functions lead to age-related neurodegenerative diseases. The impairment of mitochondrial function leads to reduced number of mitochondria, impaired mitochondrial biogenesis, and reduced capacity for oxidative phosphorylation [95]. Therefore, to evaluate the impact of PtNPs and RA on the number of mitochondria, we performed reverse transcription-quantitative polymerase chain reaction (RT-qPCR) for quantitatively analyzing the number of mitochondria. The results showed that the combination of PtNPs and RA significantly decreased the number of mitochondria by up to 5 folds, compared with the control. Moreover, SH-SY5Y cells treated with PtNPs, RA, or cisplatin exhibited 1-fold decrease in the number of mitochondria, compared with the untreated cells (Figure 8D). SH-SY5Y cells treated with AgNPs showed increased size and decreased number of mitochondria [96]. The combination of AgNPs and HDAC inhibitors showed reduced mitochondrial level in human alveolar basal epithelial cells [63].

Mitochondrial biogenesis is crucial to the maintenance of the number and size of mitochondria, involving both the nuclear and the mitochondrial genomes, and it is regulated by various transcription factors. Particularly, peroxisome proliferator-activated receptor (PPAR)-γ coactivator-1α (PGC-1α) is a co-transcriptional regulation factor that regulates the process of mitochondrial biogenesis by interacting with many transcription factors [97]. Therefore, to determine the effect of the combination of PtNPs and RA on PGC-1α expression, we treated SH-SY5Y cells with PtNPs, RA, PtNPs and RA, or cisplatin for 24 h, and measured the expression of PGC-1α by using RT-qPCR. The results showed that the combination of PtNPs and RA significantly decreased the expression of PGC-1α by up to 8 folds, compared with the control, whereas PtNPs, RA, and cisplatin decreased the expression by 4, 2, and 6 folds, respectively, compared with the untreated cells (Figure 8E). Collectively, the results suggest that the combination of PtNPs and RA potentially induced mitochondrial dysfunction by altering MMP, inhibiting the synthesis of ATP, and reducing the number of mitochondria and biogenesis.

### 2.9. PtNPs and RA Induce ERS and Apoptosis

**Figure 9:**
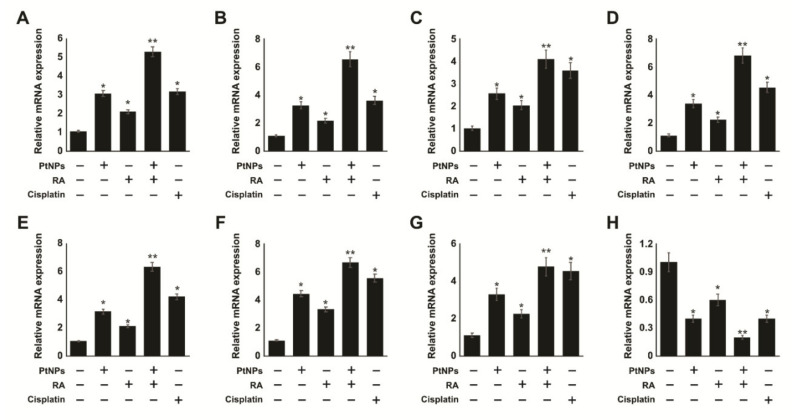
PtNPs and RA enhance the expression of endoplasmic reticulum stress (ERS) and apoptotic genes. SH-SY5Y cells were incubated with PtNPs (25 μg/mL), RA (12.5 μM), combination of PtNPs (25 μg/mL) and RA (12.5 μM), or cisplatin (6.5 μM) for 24 h. The mRNA expression of ERS-related genes, IRE1 (**A**), PERK (**B**), ATF6 (**C**), and ATF4 (**D**) was analyzed using quantitative reverse-transcription polymerase chain reaction. mRNA expression of apoptotic genes, p53 (**E**), Bax (**F**), caspase-3 (**G**), and Bcl-2 (**H**). The fold change in the expression was determined relative to *GAPDH* expression. The results are expressed as mean fold change ± standard deviation from three independent experiments. There was a significant difference between treated cells and untreated cells per Student’s *t*-test (* *p* < 0.05). The treated groups showed statistically significant differences from the control group per the Student’s *t*-test; * *p* < 0.05 was considered significant, ** *p* < 0.01 was considered highly significant. PtNPs, platinum nanoparticles; RA, retinoic acid; IRE1, inositol-requiring enzyme 1; PERK, protein kinase-like ER kinase; ATF6, activating transcription factor 6; ATF4, activating transcription factor 4.

Endoplasmic reticulum stress (ERS) may initiate the unfolded protein response (UPR) to restore cellular homeostasis or induce apoptosis [98]. UPR is regulated by various transmembrane proteins such as protein kinase-like ER kinase (PERK), inositol-requiring enzyme 1 (IRE1), activating transcription factor (ATF6), and ATF4, which are involved in the maintenance of homoeostasis. Therefore, to determine whether the administration of PtNPs and RA caused ERS, the mRNA expression of genes associated with ERS, which included PERK, IRE1, ATF6, and ATF4, were analyzed by RT-qPCR. Our results demonstrated that the mRNA expression levels of PERK, IRE1, ATF6, and ATF4 in SH-SY5Y cells treated with PtNPs and RA for 24 h were significantly higher by 2–4 fold than those in the control (Figure 9). Similarly, SH-SY5Y cells treated with PtNPs, RA, and cisplatin exhibited significantly higher expression of the genes than the untreated cells; however, the expression levels were lower than those observed with the combination of PtNPs and RA. ZnONPs increased the phosphorylation of RNA-dependent PERK, and eukaryotic initiation factor 2α decreased the protein translation and synthesis [99]. Previous studies have suggested that ERS can be induced by various types of nanoparticles. These findings suggest that PtNPs induce apoptosis through the induction of ERS. The activation of PERK, IRE1, ATF6, and ATF4 in SH-SY5Y cells by PtNPs was consistent with the results of previous studies performed using various nanoparticles, such as those of titanium dioxide [100], zinc oxide [99], silver [101], and silica [102].

Furthermore, we investigated whether ERS is involved in PtNP and RA-induced apoptosis of SH-SY5Y cells by measuring the expression levels of apoptotic-related genes, including p53, Bax, caspase-3, and Bcl-2. Our results showed that the mRNA expression levels of pro-apoptotic genes, p53, Bax, and caspase-3 were significantly higher than those in the control (Figure 9). PtNP and RA treatment resulted in significant decrease in the mRNA expression of the anti-apoptotic gene, Bcl-2, in SH-SY5Y cells, and although PtNP, RA, and cisplatin treatment induced the expression of pro-apoptotic genes, the induction level was lower than that with the combination treatment. CHOP/GADD153, a proapoptotic protein, is involved in the promotion of apoptosis through the PERK/ATF4 and ATF6 pathways [103]. Under ERS, IRE1 induces apoptosis by increasing the expression levels of ER chaperons (grp78, grp94, and pdi-3) and activating JNK [104]. CHP134 and NB-39-nu cells treated with RA showed significant downregulation of Bcl-2, which was associated with the significant activation of caspase-9 and caspase-3 as well as cytoplasmic release of cytochrome *c* from the mitochondria in a p53-independent manner [105]. Giorgi et al. demonstrated that p53 at the endoplasmic reticulum and the mitochondria-associated membranes, interacting with sarco/ER Ca^2+^-ATPase pumps, modulates ER–mitochondria cross-talk and Ca^2+^-dependent apoptosis [106]. RA activates p53 and Bax, which is required for initiation of mitochondrial-mediated apoptosis pathway in human pluripotent stem cells (Setoguchi et al., 2016). Human umbilical vascular endothelial cells exposed to thapsigargin treatment showed increased levels of cyt c, caspase-3, caspase-4, caspase-9, caspase-12, and PARP through the activation of ATF6. Furthermore, the over-expression of ATF6 increases the expression of CHOP, and JNK/NF-κB was also involved in ERS-induced cell apoptosis, which supports that ERS activates mitochondrion-mediated apoptosis [107]. RA was also reported to significantly activate the mRNA expression of p53, p21, Bax, Bak, caspase-3, and caspase-9, and decrease the expression of Bcl-2 and Bcl-Xl in F9 teratocarcinoma stem cells [66]. In this study, the combination of the two agents resulted in a more effective and efficient induction of programmed cell death in SH-SY5Y cells. Altogether, these findings indicate that the combination of PtNPs and RA induces ERS-mediated cell apoptosis.

### 2.10. PtNPs and RA Induce Apoptosis and Oxidative DNA Damage

**Figure 10:**
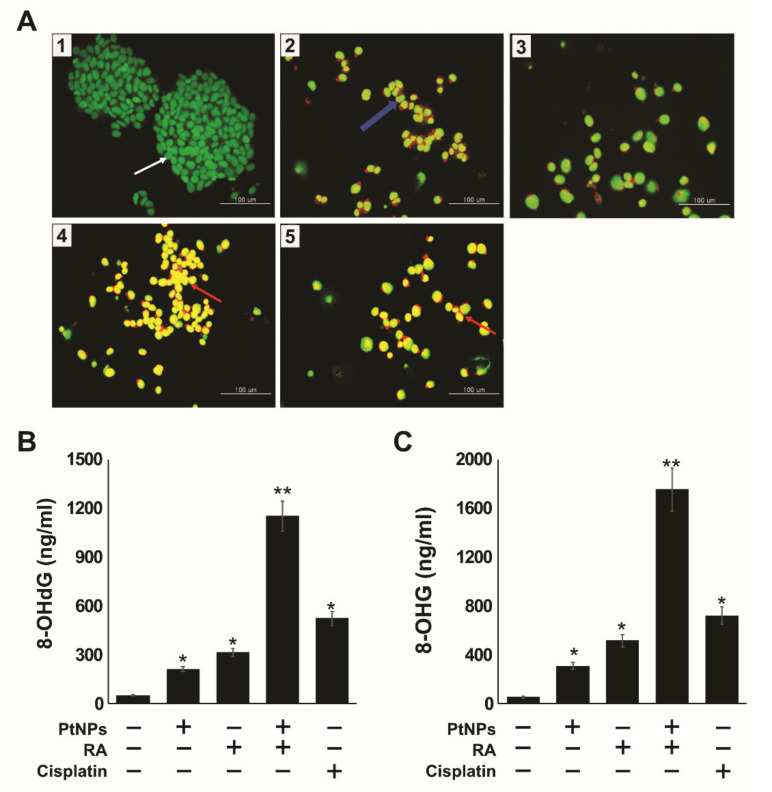
PtNPs and RA induce apoptosis and accumulation of 8-OHdG and 8-OHG.SH-SY5Y cells were incubated with PtNPs (25 μg/mL), RA (12.5 μM), combination of PtNPs (25 μg/mL) and RA (12.5 μM), or cisplatin (6.5 μM) for 24 h. Morphologic observation with acridine orange/ethidium bromide (AO/EB) staining (**A**). SH-SY5Y cells were treated without (1), with PtNPs (2), RA (3), PtNPs and RA (4), or cisplatin (5) for 24 h. White color arrow indicates viable cells, blue color indicates early apoptotic cells, and red color indicates late apoptotic cells. Each experiment was performed in triplicate (*n* = 3). Original magnification was 200 μm. The level of 8-OHdG was measured by ELISA after 24 h of exposure (**B**). The level of 8-OHG was measured by ELISA after 24 h of exposure (**C**). The results are expressed as mean ± standard deviation from three independent experiments. There was a significant difference between treated cells and untreated cells per Student’s *t*-test (* *p* < 0.05). The treated groups showed statistically significant differences from the control group per the Student’s *t*-test; * *p* < 0.05 was considered significant, ** *p* < 0.01 was considered highly significant. PtNPs, platinum nanoparticles; RA, retinoic acid; ELISA, enzyme-linked immunosorbent assay; 8-OHdG, 8-hydroxy-2-deoxyguanosine; 8-OHG, 8-hydroxyguanosine.

To confirm that apoptosis was induced by PtNPs and RA, SH-SY5Y cells were analyzed in the presence of acridine orange/ethidium bromide (AO/EB) stain. The different group of cells, including the control cells, were cultured in DMEM and stained with AO/EB. Cells treated with PtNPs, RA, PtNPs and RA, and cisplatin exhibited significant apoptosis; however, the combination of PtNPs and RA induced more pronounced effect than the single treatments (Figure 10A). Green-stained cells represented viable cells, whereas yellow-stained cells represented early apoptotic cells, and red- or orange-stained cells represented late apoptotic cells. SH-SY5Y cells treated with PtNPs and RA showed significant changes in cellular morphology, including chromatin condensation, membrane blebbing, and fragmented nuclei, which indicates that PtNPs and RA potentially induced early and late apoptosis. Similar features were also observed in cells treated with PtNPs, RA, and cisplatin. In addition, the combination treatment caused severe damage and late-stage apoptosis with the presence of apoptotic bodies. Cells treated with PtNPs, RA, PtNPs and RA, and cisplatin were more prone to chromatin condensation, nuclear fragmentation, and apoptosis, compared with the control. Several studies have reported that metallic nanoparticles such as silver, palladium, and platinum can potentially induce apoptosis via DNA chromatin condensation and fragmentation in various type of cancer cells, including human breast cancer cells [108], ovarian cancer cells [109], human neuroblastoma cancer cells, [110] and human bone OS epithelial cells [49].

Oxidative stress-induced DNA and RNA damage include oxidized DNA bases as well as DNA single strand and double strand breaks. Guanine is the most susceptible to oxidation by ROS, resulting in the generation of 8-oxoguanine [111]. Thus, we examined the levels of 8-hydroxy-2-deoxyguanosine (8-OHdG) and 8-hydroxyguanosine (8-OHG) in SH-SY5Y cells exposed to PtNPs, RA, PtNPs and RA, and cisplatin for 24 h, and the results revealed that the accumulation rate of 8-OHdG (1100 ng/mL) was significantly higher, compared with that of either PtNPs (200 ng/mL) or RA treatment alone (300 ng/mL). However, the accumulation of 8-OHdG was remarkably higher (1700 ng/mL) in cells treated with PtNPs and RA than in those treated singly with either PtNPs (300 ng/mL) or RA (500 ng/mL) (Figure 10B,C). For example, DNA damage was induced in BALB/c 3T3 fibroblasts exposed to gold nanoparticles through an indirect mechanism triggered by oxidative stress [112], and severe oxidative damage to DNA and accumulation of 8-OHdG and 8-OHG SH-SY5Y were observed in cells exposed to silver nanoparticles [49]. It has also been reported that TiO_2_NP induced ROS-mediated oxidative stress, the activation of p53, Bax, and caspase- 3, and oxidative DNA damage in HEK-293 cells [113]. Similarly, human hepatocytes and embryonic kidney cells exposed to zinc oxide nanoparticles exhibited altered cellular morphology, mitochondrial dysfunction, increased levels of oxidative stress markers, and oxidative DNA damage [114]. HaCaT cells treated with silicon dioxide NPs exhibited increased level of ROS, DNA damage, and apoptosis in size- and concentration-dependent manners [115]. Similarly to other metallic NPs, PtNPs induced oxidative DNA damage in human cancer cells, including human monocytic THP-1 cells [48]. The combination of PtNPs and doxorubicin caused significant oxidative DNA damage and induced the accumulation of 8-OHdG and 8-OHG in osteosarcoma cancer cells [49]. Altogether, these results clearly support that the combination of PtNPs and RA can potentially induce DNA damage.

### 2.11. Combination of PtNPs and RA Increases Differentiation and the Expression of Differentiation Markers in SH-SY5Y Cells

**Figure 11:**
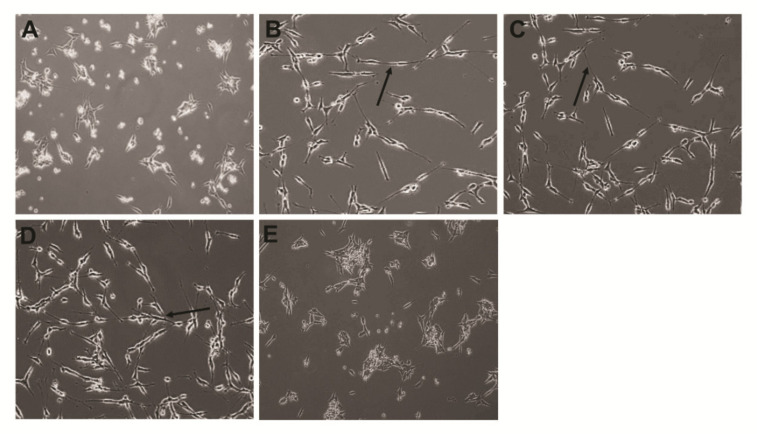
(**A**). PtNPs and RA enhance the differentiation and expression of differentiation markers of SH-SY5Y cells. SH-SY5Y cells were incubated with PtNPs (25 μg/mL), RA (12.5 μM), combination of PtNPs (25 μg/mL) and RA (12.5 μM), or cisplatin (6.5 μM) for 24 h. Phase contrast microscopy images showing the morphological changes in SH-SY5Y cells after treatment without (**A**) and with PtNPs (**B**), RA (**C**), PtNPs and RA (**D**), and cisplatin (**E**) in 1% serum-supplemented medium. The black arrows indicate significant, lengthy neurite outgrowth. At least three independent experiments were performed for each sample. Scale bar is 100 µm.

To investigate the potential ability of PtNPs and RA to induce differentiation of SH-SY5Y cells, the cells were treated with 1% FBS for 24 h, and the medium was removed afterwards. Next, the cells were incubated with fresh medium containing PtNPs, RA, PtNPs and RA, or cisplatin for another 24 h. Differentiation was assessed by phase contrast microscopy. As shown in Figure 11, the phase contrast images of SH-SY5Y in the untreated and cisplatin-treated cultures showed no neurite outgrowth, and the cells had a round shape and protrusion, whereas PtNPs- or RA-treated cells showed significant number of long neurites (black arrows). Similarly, cells treated with the combination of PtNPs and RA had significantly larger number of, more clustered, and branched longer neuritis, compared with the untreated cells. However, the untreated cells were more numerous, more clustered, longer, and with more detectable network formation than the PtNPs- or RA-induced neurites. The morphology of SH-SY5Y cells treated with PtNPs and RA was relatively polar, and the cells grew faster and significantly longer than cells treated with PtNPs or RA. These findings corroborated with those of a recent study on SH-SY5Y cells, which used various differentiating agents, including RA [116] and a combination of RA and brain-derived neurotrophic factor (BDNF) [117].

SH-SY5Y cells treated with estradiol, RA, and cholesterol showed different ranges of branching of shorter and longer neurites and detectable network formation [118]. RA is a well-known inducer of differentiation and proliferation in normal stem cells. Similarly, metallic nanoparticles, such as silver, induce differentiation in SH-SY5Y cells [119] and F9 teratocarcinoma stem cells [120]. Treatment of breast cancer stem-like cells, MCF7/C6, with ATRA caused cell differentiation, reduced invasion and migration, and increased sensitivity to anticancer treatment [121]. In a recent study, graphene induced significant differentiation in human neural stem cells [122] and SH-SY5Y cells [78]. AgNPs-induced oxidative stress triggered neuronal differentiation through the modulation of phosphatases and the kinase signaling pathways in SH-SY5Y cells [119]. Similarly, in this study, PtNPs and RA induced oxidative stress through the production of ROS and consequently induced differentiation in SH-SY5Y cells.

### 2.12. Effect of Cisplatin on Undifferentiated and Differentiated SH-SY5Y Cells

**Figure 12:**
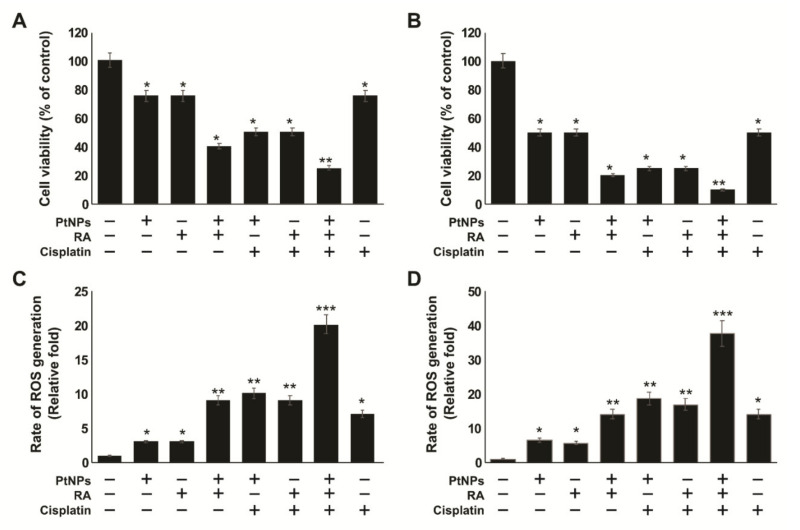
Effect of cisplatin on undifferentiated and differentiated SH-SY5Y cells. SH-SY5Y cells were pre-incubated with PtNPs (25 μg/mL), RA (12.5 μM), or the combination of PtNPs (25 μg/mL) and RA (12.5 μM) for 24 h. After preincubation, cisplatin (6.5 μM) was added to the cells. Cell viability was determined in undifferentiated SH-SY5Y cells (**A**) and differentiated SH-SY5Y cells (**B**). Determination of ROS using DCFH-DA in undifferentiated SH-SY5Y cells (**C**) and differentiated SH-SY5Y cells (**D**). The results are expressed as mean ± standard deviation from three independent experiments. There was a significant difference between treated cells and untreated cells per Student’s *t*-test (* *p* < 0.05). The treated groups showed statistically significant differences from the control group per the Student’s *t*-test; * *p* < 0.05 was considered significant, ** *p* < 0.01 was considered highly significant, and *** *p* < 0.001 was considered very highly significant. PtNPs, platinum nanoparticles; RA, retinoic acid; DCFH-DA, dichloro-dihydro-fluorescein diacetate.

Differentiation is one of the best approaches that can be used to eliminate cancer stem cells [123]. Therefore, development of new approaches targeting at the differentiation process is essential in anticancer therapy. As differentiated cells exhibit better responsiveness to drugs than undifferentiated cells, we evaluated the sensitivity of SH-SY5Y cells to cisplatin. We used PtNPs, RA, and cisplatin at their IC _25_ to avoid major cytotoxic effects. To determine the effect of cisplatin on undifferentiated and differentiated SH-SY5Y cells, both forms of cells were pretreated with PtNPs and RA and subsequently treated with cisplatin for 24 h. Results showed that the treatment of differentiated SH-SY5Y cells with both PtNPs and RA in the presence of cisplatin or with PtNPs or RA in the presence of cisplatin, resulted in stronger cytotoxic effects than those in the absence of cisplatin in either the undifferentiated or differentiated cells. Moreover, the combination of PtNPs and RA in the presence of cisplatin exerted a more remarkable effect than either PtNPs or RA in the presence of cisplatin (Figure 12A,B). This result suggests that combination therapy with differentiated cells might be valuable for improving the outcome of patients with high-risk neuroblastoma. In addition, our results support the potential benefits of combination therapy using PtNPs and RA in neuroblastoma cancer. ATRA effectively induced the differentiation of tumor-initiating cells, which potentiated the cytotoxic effects of cisplatin. The combinatorial treatment with ATRA acid and cisplatin reduced protein kinase B (Thr308) phosphorylation and promoted the apoptosis of hepatocellular carcinoma cells more significantly than treatment with cisplatin alone [124]. Similarly, we have reported significant effect of cisplatin on silver nanoparticles-differentiated F9 cells. Cisplatin significantly inhibited the proliferation of differentiated F9 cells after 24-h treatment with AgNPs [123]. Our findings suggest that PtNPs potentiated the effect of cisplatin on the viability of SH-SY5Y cells, indicating that PtNPs have the capacity to re-sensitize cells to cisplatin cytotoxicity by directly/indirectly targeting cancer stem cells. Hence, the data revealed that the combination of PtNPs and RA in the presence of cisplatin could increase the efficacy against cancer stem-like cells.

Excessive production of ROS leads to damage to various macromolecules such as DNA, proteins, and lipids, which in turn leads to progressive cell dysfunction and apoptosis. The differentiated neuronal cells are highly susceptible to oxidant-mediated damage due to differences arising in the relative activities of essential antioxidant enzymes and the limited ability to generate or recycle GSH [125]. To investigate the influence of cisplatin on SH-SY5Y cells differentiated with PtNPs and RA, both the differentiated and undifferentiated cells were challenged with cisplatin for 24 h. Intracellular levels of ROS were detected by DCFA-DA, and our results showed that differentiated cells exhibited significant elevation of ROS, compared with the control and undifferentiated cells. The level of ROS production was several folds higher in cells treated with PtNPs and RA in the presence of cisplatin (Figure 12C,D). These results indicate that the oxidative properties of PtNPs, RA, and cisplatin induce the production of intracellular ROS during neuronal differentiation. This may be due to the apoptotic effect of PtNPs, RA, and cisplatin in neuronal cells, whereby they significantly induce ROS-medicated cellular toxicity and oxidative stress in differentiated cells compared with undifferentiated cells or untreated cells. Previous studies have suggested that excessive production of ROS in differentiated cells induces apoptosis, increases lactate production, and leads to an uncoupling of electron transport chain flux from ATP production in embryonic stem cells. This is associated with immature mitochondrial morphology and reduced redox environment [126,127]. Furthermore, forced activation of oxidative phosphorylation leads to loss of stem cell properties and increased differentiation or apoptosis [127]. Collectively, our findings suggest that treatment with the combination of PtNPs and RA in the presence of cisplatin causes excessive production of ROS, which could modulate various intracellular signaling pathways in differentiated cells.

**Figure 13:**
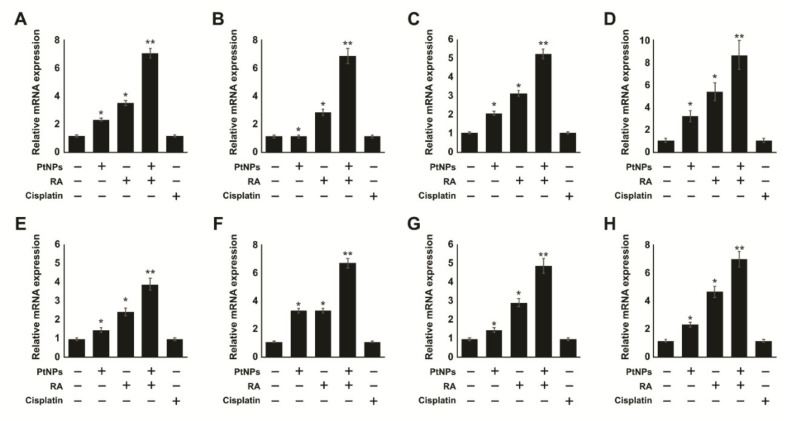
Effect of cisplatin on undifferentiated and differentiated SH-SY5Y cells. SH-SY5Y cells were pre-incubated with PtNPs (25 μg/mL), RA (12.5 μM), or the combination of PtNPs (25 μg/mL) and RA (12.5 μM) for 24 h. After preincubation, cisplatin (6.5 μM) was added to the cells. We examined the expression of various neuronal markers of differentiation, including MAP2 (**A**), beta-tubulin III (**B**), neurogenin (**C**), GAP-43 (**D**), DRD2 (**E**), neurofascin (**F**), neuropilin 1 (**G**), and gamma neuronal (**H**) in SH-SY5Y cells exposed for 24 h. The relative expression levels of all the genes were increased by 4–8 folds after treatment with PtNPs and RA. The results are expressed as mean ± standard deviation from three independent experiments. There was a significant difference between treated cells and untreated cells per Student’s *t*-test (* *p* < 0.05). The treated groups showed statistically significant differences from the control group per the Student’s *t*-test; * *p* < 0.05 was considered significant, ** *p* < 0.01 was considered highly significant.

Given that RA can influence differentiation, we determined whether its combination with PtNPs could enhance the differentiation of neuronal markers. We examined the expression of various neuronal markers of differentiation, including microtubule-associated protein 2 (MAP2), beta-tubulin III, neurogenin, growth associated protein 43 (GAP-43), dopamine receptors type 2 (DRD2), neurofascin (NFASC), neuropilin 1 (NRP1), and gamma neuronal (NSE) in SH-SY5Y cells exposed for 24 h. The relative expression levels of all the genes were increased by 4–8 folds after treatment with PtNPs and RA. Moreover, the combination of PtNPs and RA upregulated all the genes more effectively than PtNPs or RA. This suggests that PtNPs could promote neuronal differentiation response to RA in SH-SY5Y cells. Interestingly, all the test genes were upregulated by 4–8 folds in the presence of PtNPs and RA (Figure 13). This demonstrates that PtNPs has the capacity to enhance RA-induced neuronal gene expression. It has been reported that other nanomaterials such as silver and graphene oxide have the ability to induce the expression of various neuronal differentiation markers in SH-SY5Y cells [78,119]. Moreover, the combination of PtNPs and RA upregulated all the genes more effectively than PtNPs or RA. This suggests that PtNPs could promote neuronal differentiation response to RA in SH-SY5Y cells. Interestingly, all the test genes were upregulated by 4–8 folds in the presence of PtNPs and RA (Figure 13). This demonstrates that PtNPs has the capacity to enhance RA-induced neuronal gene expression. It has been reported that other nanomaterials such as silver and graphene oxide have the ability to induce the expression of various neuronal differentiation markers in SH-SY5Y cells [78,119].

## 3. Materials and Methods

### 3.1. Synthesis and Characterization of PtNPs

PtNPs synthesis and characterization was performed as described earlier [48]. PtNPs were synthesized through the reduction of PtCl_6_ 2- ions into PtNPs by mixing 10 mL of 1 mg/mL beta carotene with 90 mL of 1 mM aqueous H_2_PtCl_6_.6H_2_O (Sigma-Aldrich, St.Loius, MO, USA). The mixture was maintained at 100 °C (on a hotplate) in a sealed flask to avoid evaporation for 1 h, as temperature catalyzes the reduction process.

### 3.2. Cell Viability and Cell Proliferation Assay

SH-SY5Y human neuroblastoma cells were obtained from the American Type Culture Collection. Cell viability was measured using a cell counting kit-8 (CCK-8; CK04-01, Dojindo Laboratories, Kumamoto, Japan). Briefly, the cells were plated in 96-well flat-bottom culture plates containing various concentrations of PtNPs (20–100 µg/mL), RA (10–50 µg/mL) and cisplatin (5–25 µg/mL). After 24-h culture at 37 °C in a humidified 5% CO_2_ incubator, CCK-8 solution (10 μL) was added to each well, and the plate was incubated for another 2 h at 37 °C. Absorbance was measured at 450 nm using a microplate reader (Multiskan FC, Thermo Fisher Scientific Inc., Waltham, MA, USA). Cell proliferation was determined according to the manufacturer’s instructions (Roche, Belmont, CA, USA).

### 3.3. Cell Morphology Analysis

SH-SY5Y cells were plated in 6-well plates (2 × 10^5^ cells per well) and incubated with IC_25_ of PtNPs (25 μg/mL), RA (12.5 μM), PtNPs and RA (25 µg/mL + 12.5 µM), or cisplatin (6.5 μM) for 24 h. Untreated cells and cisplatin-treated cells were used as the controls. The morphology of the cells was examined with an OLYMPUS IX71 microscope (Tokyo, Japan) using the appropriate filter sets.

### 3.4. Cytotoxicity Assays

The membrane integrity of SH-SY5Y cells was evaluated using a LDH cytotoxicity detection kit (Sigma-Aldrich, St.Loius, MO, USA). A dead-cell protease activity assay was performed according to the method described earlier [34].

### 3.5. Determination of the Levels of Reactive Oxygen Species (ROS), Malondialdehyde (MDA), Nitric Oxide (NO), and Protein Carbonyl Content (PCC)

ROS, MDA, NO, and PCC was estimated as described previously [48]. Oxidative stress markers detections kits were obtained from Sigma-Aldrich, St. Louis, MO, USA. SH-SY5Y cells were treated with IC_25_ concentrations of PtNPs (25 μg/mL), RA (12.5 μM), PtNPs and RA (25 µg/mL + 12.5 µM), or cisplatin (6.5 μM) for 24 h. Detailed methodology given as Supplementary File.

### 3.6. ELISA

Levels of 4-hydroxynonenal (4-HNE), 8-hydroxy-2′-deoxyguanosine (8-OHdG), and 8-hydroxyguanosine (8-OHG) in SH-SY5Y cells exposed to PtNPs (25 μg/mL), RA (12.5 μM), PtNPs and RA (25 µg/mL + 12.5 µM), or cisplatin (6.5 μM) for 24 h were measured according to a previously described method [128]. An ELISA kit was used to measure the concentrations of 4-HNE, 8-OHdG, and 8-OHG according to the manufacturers’ instructions. ELISA kits were used to measure concentrations of 4-hydroxynonenal (4-HNE, Cusabio Biotech Co. Ltd., Wilmington, Del., USA), 8-OHdG and 8-OHG ELISA kit purchased from Trevigen, Gaithersburg, MD, USA).

### 3.7. Measurement of the Levels of Anti-Oxidative Markers

The expression levels of oxidative and anti-oxidative stress markers were determined as previously described [48]. Antioxidants kits were purchased from Sigma, St.Louis, MO, USA.

### 3.8. Determination of Mitochondrial Dysfunctions

Mitochondrial membrane potential (MMP) was measured according to the manufacturer’s instructions (Molecular Probes, Eugene, OR, USA) using the cationic fluorescent indicator, JC-1 (Molecular Probes). SH-SY5Y cells were pretreated with or without PtNPs (25 μg/mL), RA (12.5 μM), combination of PtNPs and RA (25 µg/mL + 12.5 µM), or cisplatin (6.5 μM) for 24 h, followed by incubation with 10 μM JC-1 at 37 °C for 15 min, washing with PBS, re-suspension in PBS, and measurement of the fluorescence intensity. MMP is expressed as the ratio of the fluorescence intensity of the JC-1 aggregates to that of the monomers.

The ATP level was measured according to the manufacturer’s instructions (Catalog Number MAK135, (Sigma-Aldrich, St. Louis, MO, USA). Decreased ATP levels indicated increased cytotoxicity to the treated cells.

Mitochondrial dysfunction analysis was carried out by assessing the mitochondria copy number using RT-qPCR analysis [96].

### 3.9. Determination of Apoptosis Using AO/EB Staining

For detection of apoptotic cells, SH-SY5Y cells were treated with or without PtNPs (25 μg/mL), RA (12.5 μM), PtNPs and RA (25 µg/mL + 12.5 µM), or cisplatin (6.5 μM) for 24 h. Approximately 1 μL of a dye mixture containing AO and EtBr was mixed with 9 mL of cell suspension (1 × 10^5^ cells per ml) on clean microscope cover slips. The cells were collected, washed with PBS (pH 7.2), and stained with 1 mL of AO/EtBr. After incubation for 2 min, the cells were washed twice with PBS (5 min each) and visualized under a fluorescence microscope at 400× magnification with an excitation filter at 480 nm.

### 3.10. RT-qPCR

RT-qPCR was performed according to a previously described method [129]. The sequences of the RT-qPCR primers are shown in Supplementary Tables S1 and S2.

### 3.11. Statistical Analysis

Independent experiments were repeated at least thrice, and all data are presented as mean ± standard deviation for all triplicates within an individual experiment. If a single concentration of a drug was assessed, the Student’s *t*-test was used to identify drugs that exhibited a significant effect, compared with the vehicle control group. If multiple concentrations of a drug were assessed, ANOVA was first used to assess whether there were any differences in the mean among the various concentrations of each compound, if a significant difference was determined (*p* < 0.05), Dunnett’s test, which is appropriate for multiple pair-wise comparisons against a control, was then performed.

## 4. Conclusions

Neuroblastoma is the most common extracranial solid tumor that preferentially occurs in childhood. Although the introduction of several antitumor agents and treatments has improved the survival of neuroblastoma patients, the disease is still associated with high mortality. Moreover, long term treatment leads to short- and long-term undesired toxicities. Nanoparticle-mediated combination therapy is an alternative therapeutic approach that can be used to overcome some of the common limitations of conventional chemotherapy. However, the therapeutic effects of the combination of platinum nanoparticles and RA in neuroblastoma have remained elusive. Thus, we investigated the effect of the combination of PtNPs and RA on growth inhibition, cell morphology, cytotoxicity, oxidative stress, mitochondrial dysfunction, ERS, apoptosis, oxidative DNA damage, and differentiation in human neuroblastoma cancer cells (SH-SY5Y). PtNPs acted synergistically with RA to induce cell death, which was associated with growth inhibition, loss of cell viability, loss of mitochondrial membrane potential, activation of ERS genes, pro-apoptotic genes, and caspase-3, and eventually DNA damage. In the present study, we demonstrated that treatment of differentiated SH-SY5Y cells with PtNPs and RA in the presence of cisplatin exhibited significant and better cytotoxic effects than that of undifferentiated cells. Therefore, PtNPs and RA could work together to exert enhanced therapeutic efficacy and minimal cytotoxic side-effects. The present study provided experimental evidence of the potential benefit of the combination of PtNPs and RA in the prevention and treatment of neuroblastoma. Furthermore, these findings suggest that PtNPs and RA acted synergistically to induce apoptosis and may have potential for combination biotherapy in the treatment of malignant diseases such as neuroblastoma. However, further studies with animal models are warranted to decipher the molecular mechanisms associated with the synergistic actions of PtNPs and RA.

## Supplementary Materials

Supplementary materials can be found at https://www.mdpi.com/1422-0067/21/18/6792/s1.
