# Supplementary material for: Anticancer Properties of Platinum Nanoparticles and Retinoic Acid: Combination Therapy for the Treatment of Human Neuroblastoma Cancer
Source: Int J Mol Sci. 2020 Sep 16;21(18):6792. doi: 10.3390/ijms21186792 (PMC7554966; doi:10.3390/ijms21186792)
Supplement: Supplementary file 1 [file ijms-21-06792-s001.pdf]

International Journal of *Molecular Sciences*

MDPI

Supplementary files–Materials and Methods

Cell Lines and Reagents

Beta carotene as well as dried and hydrated hexachloroplantinic acid ($H_2PtCl_6{\cdot}6H_2O$) were purchased from Sigma-Aldrich (St. Louis, MO, USA). Penicillin-streptomycin, trypsin-EDTA, DMEM, fetal bovine serum (FBS), and antibiotic-anti-mycotic reagents were obtained from Life Technologies/Gibco (Grand Island, NY, USA). An in vitro toxicology assay kit, reagent kits for the measurement of MDA and PCC, and antioxidant assay kits were purchased from Sigma-Aldrich (St. Louis, MO, USA). All other chemicals were purchased from Sigma-Aldrich, unless otherwise stated.

Synthesis and Characterization of PtNPs

PtNPs synthesis and characterization was performed as described earlier [48]. PtNPs were synthesized through the reduction of $PtCl_6$ 2- ions into PtNPs by mixing 10 mL of 1 mg/mL beta carotene with 90 mL of 1 mM aqueous $H_2PtCl_6.6H_2O$. The mixture was maintained at 100 °C (on a hotplate) in a sealed flask to avoid evaporation for 1 h, as temperature catalyzes the reduction process. For the control experiments, identical amounts of platinum solution and beta carotene were maintained separately under the same reaction conditions. The reduced platinum solution was sonicated for 10 min to separate platinum nanoparticles from the biomolecules. After sonication, the solution was filtered with a 0.2-μm syringe filter. The reduced platinum metal was purified by repeated centrifugation at 5,000 rpm for 30 min, and the pellets were washed with distilled water to remove the impurities. Purified PtNPs were characterized using various analytical techniques.

Cell Culture and Treatments

The human neuroblastoma cell line SH-SY5Y was obtained from ATCC. SH-SY5Y cells were cultured in DMEM containing 10% FBS, 100 U/mL penicillin, and 100 mg/mL streptomycin and incubated in a humidified atmosphere with 5% CO_2 at 37 °C. All the cells were cultured in 75-cm^2 tissue culture flasks (Corning, NY, USA) at 37 °C in the presence of 5% CO_2 and 95% relative humidity. The cells were sub-cultured, usually twice a week, with 1×10^6 viable cells/ml and incubated at 37 °C in a 5% CO_2 atmosphere. The medium was replaced the next day with fresh media, and the cells were further incubated for 24 h prior to treatment with PtNPs or RA or combination of PtNPs and RA. The experiments were performed in 96-, 24-, and 12-well plates and 100-mm cell culture dishes, as required. For all the differentiation experiments, SH-SY5Y cells were grown in the same culture media supplemented with 1% FBS for 24 h, after which they were washed with fresh media and further incubated with IC_{25} concentrations of PtNPs (25 μg/mL), RA (12.5 μM), PtNPs and RA (25 μg/mL + 12.5 μM), or cisplatin (6.5 μM) for another 24 h.

Cell Viability Assay

Cell viability was measured using a cell counting kit-8 (CCK-8; CK04-01, Dojindo Laboratories, Kumamoto, Japan). Briefly, the cells were plated in 96-well flat-bottom culture plates containing various concentrations of PtNPs, RA, or combination of PtNPs and RA. After 24-h culture at 37 °C in a humidified 5% CO_2 incubator, CCK-8 solution (10 μl) was added to each well, and the plate was incubated for another 2 h at 37 °C. Absorbance was measured at 450 nm using a microplate reader (Multiskan FC; Thermo Fisher Scientific Inc., Waltham, MA, USA).

BrdU Cell Proliferation Assay

Cell proliferation was determined according to the manufacturer's instructions (Roche). SH-SY5Y cells were treated with various concentrations of PtNPs, RA, or a combination of PtNPs and RA for 24 h, followed by the addition of BrdU labeling solution to the culture medium and incubation for 2 h. The cells were fixed and the level of incorporated BrdU was determined using the BrdU ELISA

kit (Roche) according to the manufacturer's instructions. Proliferation of the untreated cells at 0 h was considered 100%.

Cell Morphology Analysis

SH-SY5Y cells were plated in 6-well plates (2×10^5 cells per well) and incubated with IC_{25} of PtNPs (25 µg/mL), RA (12.5 µM), PtNPs and RA (25 µg/mL + 12.5 µM), or cisplatin (6.5 µM) for 24 h. Untreated cells and cisplatin-treated cells were used as the controls. The morphology of the cells was examined with an OLYMPUS IX71 microscope (Tokyo, Japan) using the appropriate filter sets.

Assessment of Membrane Integrity

The membrane integrity of SH-SY5Y cells was evaluated using a LDH cytotoxicity detection kit. Briefly, SH-SY5Y cells were treated with or without IC_{25} of PtNPs(25 µg/mL), RA (12.5 µM), PtNPs and RA (25 µg/mL + 12.5 µM), or cisplatin (6.5 µM) for 24 h. Subsequently, 100 µL of cell-free supernatant from each well was transferred in triplicate into the wells of a 96-well plate, and 100 µL of the LDH reaction mixture was added to each well. After 3 h of incubation under standard conditions, the optical density of the final solution was determined at a wavelength of 490 nm using a microplate reader.

Assessment of Dead-Cell Protease Activity

A dead-cell protease activity assay was performed according to the method described earlier [34]. SH-SY5Y cells were treated with or without PtNPs, RA, or a combination of PtNPs and RA for 24 h. The protease activity was determined by assessing the association of intracellular proteases with a luminogenic peptide substrate (alanyl-alanylphenylalanyl-aminoluciferin). Luminogenic peptide substrate (5 µl) was added to each well, and luminescence was measured to determine the number of dead cells. The peptide substrate was incubated for 15 min at 37 °C, and the luminescence was measured with a luminescence counter (Perkin Elmer, Waltham, MA, USA). The degree of luminescence measured in this assay reflected the dead-cell protease activity.

Determination of the Levels of ROS, MDA, NO, and Carbonylated Protein

ROS was estimated as described previously [48]. SH-SY5Y cells were treated with IC_{25} concentrations of PtNPs (25 µg/mL), RA (12.5 µM), PtNPs, and RA (25 µg/mL + 12.5 µM), or cisplatin (6.5 µM) for 24 h. After washing twice with phosphate-buffered saline (PBS), 20 µM DCFH2-DA was added to the cells, and incubation was continued for 30 min at 37 °C. The cells were rinsed with PBS, followed by the addition of 2 mL PBS to each well and the measurement of the fluorescence intensity with a Gemini EM spectrofluorometer (Molecular Devices, Sunnyvale, CA, USA) at an excitation wavelength of 485 nm and an emission wavelength of 530 nm.

MDA levels were determined using a thiobarbituric acid-reactive substances assay as previously described [48]. NO production was spectrophotometrically quantified using Griess reagent (Sigma-Aldrich); absorbance was measured at 540 nm, and nitrite concentration was determined using a calibration curve prepared with sodium nitrite as the standard [48]. Carbonylated protein content was measured according to a previously described method [48].

ELISA

Levels of 4-HNE, 8-OHdG, and 8-OHG in SH-SY5Y cells exposed to PtNPs (25 µg/mL), RA (12.5 µM), PtNPs and RA (25 µg/mL + 12.5 µM), or cisplatin (6.5 µM) for 24 h were measured according to a previously described method [128]. An ELISA kit was used to measure the concentrations of 4-HNE, 8-OHdG, and 8-OHG according to the manufacturers' instructions.

Measurement of the Levels of Anti-Oxidative Markers

The expression levels of oxidative and anti-oxidative stress markers were determined as previously described [48]. SH-SY5Y cells were treated with or without PtNPs (25 μg/mL), RA (12.5 μM), PtNPs and RA (25 μg/mL + 12.5 μM), or cisplatin (6.5 μM) for 24 h. The levels of anti-oxidative stress markers, reduced GSH, TRX, CAT, SOD, GPx, and GST were determined according to the manufacturer's instructions. The cells were harvested in chilled PBS by scraping and washing twice with 1 × PBS at 4 °C for 6 min each at 1500 rpm. The cell pellet was sonicated at 15 W for 10 s (three cycles) to obtain the cell lysate. The resultant supernatant was stored at −70 °C until further analysis.

Determination of Mitochondrial Membrane Potential (MMP)

MMP was measured according to the manufacturer's instructions (Molecular Probes, Eugene, OR, USA) using the cationic fluorescent indicator, JC-1 (Molecular Probes). SH-SY5Y cells were pretreated with or without PtNPs (25 μg/mL), RA (12.5 μM), combination of PtNPs and RA (25 μg/mL + 12.5 μM), or cisplatin (6.5 μM) for 24 h, followed by incubation with 10 μM JC-1 at 37 °C for 15 min, washing with PBS, re-suspension in PBS, and measurement of the fluorescence intensity. MMP is expressed as the ratio of the fluorescence intensity of the JC-1 aggregates to that of the monomers.

Measurement of ATP Level

SH-SY5Y cells were treated with or without PtNPs (25 μg/mL), RA (12.5 μM), PtNPs and RA (25 μg/mL + 12.5 μM), or cisplatin (6.5 μM) for 24 h. The ATP level was measured according to the manufacturer's instructions (Catalog Number MAK135; Sigma-Aldrich). Decreased ATP levels indicated increased cytotoxicity to the treated cells.

Analysis of Mitochondrial DNA Copy Number

Mitochondrial dysfunction analysis was carried out by assessing the mitochondria copy number using RT-qPCR analysis. SH-SY5Y cells were pretreated with or without PtNPs (25 μg/mL), RA (12.5 μM), PtNPs and RA (25 μg/mL + 12.5 μM), or cisplatin (6.5 μM) for 24 h. To determine the copy number, the following primers were used for mtDNA: forward primer, CCTATCACCCTTGCCATCAT; reverse primer, AGGCTGTTGCTTGTGTGAC. To quantify the nuclear DNA, we used a primer set that detects the Pecam gene on chromosome 6 as follows: forward primer, ATGGAAAGCCTGCCATCATG; reverse primer, TCCTTGTTGTTCAGCATCAC (Gurunathan et al., 2019).

Determination of Apoptosis Using AO/EB Staining

For detection of apoptotic cells, SH-SY5Y cells were treated with or without PtNPs (25 μg/mL), RA (12.5 μM), PtNPs and RA (25 μg/mL + 12.5 μM), or cisplatin (6.5 μM) for 24 h. Approximately 1 μL of a dye mixture containing AO and EtBr was mixed with 9 mL of cell suspension (1×10^5 cells per mL) on clean microscope cover slips. The cells were collected, washed with PBS (pH 7.2), and stained with 1 mL of AO/EtBr. After incubation for 2 min, the cells were washed twice with PBS (5 min each) and visualized under a fluorescence microscope at 400× magnification with an excitation filter at 480 nm.

RT-qPCR

RT-qPCR was performed according to a previously described method [129]. SH-SY5Y cells were treated with or without PtNPs (25 μg/mL), RA (12.5 μM), PtNPs and RA (25 μg/mL + 12.5 μM), or cisplatin (6.5 μM) for 24 h. Total RNA was extracted using a PicoPure RNA isolation kit (Arcturus Bioscience, Mountain View, CA, USA). Samples were prepared according to the manufacturer's instructions. RT-qPCR was conducted using a Vill7 device (Applied Biosystems, Foster City, CA, USA) and SYBR Green was used as the double-stranded DNA-specific fluorescent dye (Applied Biosystems). The expression levels of the target genes were normalized to that of glyceraldehyde-3-phosphate dehydrogenase (*GAPDH)*, which was unaffected by the treatment. The sequences of the RT-qPCR primers are shown in Table S1 and S2.

Statistical Analysis

Independent experiments were repeated at least thrice, and all data are presented as mean ± standard deviation for all triplicates within an individual experiment. If a single concentration of a drug was assessed, the Student's *t*-test was used to identify drugs that exhibited a significant effect, compared with the vehicle control group. If multiple concentrations of a drug were assessed, ANOVA was first used to assess whether there were any differences in the mean among the various concentrations of each compound; if a significant difference was determined ($p < 0.05$), Dunnett's test, which is appropriate for multiple pair-wise comparisons against a control, was then performed. Statistical analysis and graphical representation of the data were performed using GraphPad Prism 5.0 (GraphPad Software, San Diego, CA).

Table S1. List of primers that were used for the mRNA quantification of the expression of apoptotic and anti-apoptotic gene expression.

Gene	List of Primers Used in this Study
**PGC1 alpha**	F: CAATGAATGCAGCGGTCTTA
	R: ACGTCTTTGTGGCTTTTGCT
**IRE1**	F: GACAGGCTCAATCAAATGG
	R: CGGTCAGGAGGTCAATAACA
**P53**	F: AGAGACCGTACAGAAGA
	R:CTGTAGCATGGGATCCTTT
**PERK**	F: ATTGCATCTGCCTGGTTAC
	R: GACTCCTTCCTTTGCCTGT
**Caspase-3**	F: AGGGGTCATTTATGGGACA
	R: TACACGGGATCTGTTTCTTTG
**ATF6**	F: CAGGGAGAAGGAACTTGTGA
	R: ACTGACCGAGGAGACGAGA
**Bax**	F: CGAGCTGATCAGAACCATCA
	R: GAAAAATGCCTTTCCCCTTC
**ATF4**	F: AGTCGGGTTTGGGGGCTGAAG
	R: TGGGGAAAGGGGAAGAGGTTGTAA
**Bcl-2**	F: TAAGCTGTCACAGAGGGGCT
	R: TGAAGAGTTCCTCCACCACC
**GAPDH**	F: AGGTCGGTGTGAACGGATTTG
	R: TGTAGACCATGTAGTTGAGGTCA

Table S2. List of primers that were used for the mRNA quantification of the expression of various neuronal differentiation markers.

Gene	List of Primers Used in this Study
**MAP2**	F: CATGGGTCACAGGGCACCTATTC
	R: GGTGGAGAAGGAGGCAGATTAGCTG
**NFASC**	F: TCCTGGGCAAAGCTGAAAAC
	R: AGACGGTGAGTTTCAGGGAG
**GAP-43**	F: AGAGGAACCTGAGGCTGACC
	R: GCTAGTGGGTGGGAAAGGAC
**NEUROGENIN-1**	F: GACCCTGTTTTCTCCTTCCC
	R: CCATCTATTGCCTGCTGACTAG
**DRD-2**	F: GTCATGATCTCCATCGTCTGG
	R: AATGAAGGGCACGTAGAAGG
**NRP1**	F: GAAGCACCGAGAGAACAAGG

	R: GTTGCCCTCAAAAGACTTCG
**NSE**	F: CCCAGAACTTCCCTGATTGA
	R: AAGTGGAAGACACGTGGGAC
**β-tubulin III**	F: TCTCACAAGTACGTGCCTCG
	R: CTCCGTGTAGTGACCCTTGG

© 2020 by the authors. Licensee MDPI, Basel, Switzerland. This article is an open access article distributed under the terms and conditions of the Creative Commons Attribution (CC BY) license (http://creativecommons.org/licenses/by/4.0/).
